# Genetic and genomic insights into morphological defects in nellore cattle: a comprehensive review

**DOI:** 10.1007/s11250-026-05433-8

**Published:** 2026-10-18

**Authors:** Milena Aparecida Ferreira Campos, Hinayah Rojas de Oliveira, Louise Sarmento Martins de Oliveira, Henrique Alberto Mulim, Eduarda da Silva Oliveira, Gregorio M. F. de Camargo, Raphael Bermal Costa

**Affiliations:** 1 Federal University of Bahia, School of Veterinary Medicine and Animal Science, Salvador, Bahia Brazil; 2 Department of Animal Sciences, Purdue University, West Lafayette, IN USA; 3 São Paulo State University, School of Agriculture and Veterinary Science, Jaboticabal, SP Brazil

**Keywords:** Genetic parameters, Visual scoring systems, Threshold, Linear models

## Abstract

Morphological defects in Nellore cattle, including abnormalities in conformation, feet and legs, navel structure, pigmentation, and craniofacial development, negatively impact productivity, welfare, and long-term herd sustainability. This comprehensive review aimed to synthesize existing genetic and genomic evidence on these defects in purebred Nellore cattle. Studies were identified through searches of Web of Science, PubMed, and SciELO up to September 2025. Eligible studies included genetic or genomic analyses of morphological defects in purebred Nellore cattle. The methodological characteristics and limitations of the included studies were evaluated descriptively. Results were synthesized using a narrative descriptive approach due to heterogeneity in phenotypes and methodologies. A total of 89 studies were included. This review summarizes phenotype definitions and scoring systems (e.g., MES, EPMURAS, MERCOS) used in breeding programs. Most reported heritability estimates ranged from approximately 0.10 to 0.68 across traits and analytical scales, with liability-scale estimates for binary defects reaching 0.54 for depigmentation. Genetic correlations with growth traits were generally favorable or weak, whereas evidence regarding reproductive traits was limited and less consistent. Linear models are widely used to analyze morphological defects but may underestimate genetic variance for categorical and unbalanced traits, whereas threshold models offered improved theoretical suitability at higher computational cost. Key limitations across studies included evaluator subjectivity, imbalanced score distributions, limited phenotypic granularity, and environmental heterogeneity. Morphological defects show sufficient genetic variability to support inclusion in genetic evaluations. Standardized phenotyping protocols, improved scoring strategies, and expanded genomic analyses are needed to enhance selection accuracy and animal welfare in Nellore cattle.

**Supplementary Information:**

The online version contains supplementary material available at https://doi.org/10.1007/s11250-026-05433-8.

## Introduction

Brazil holds a prominent position in the global agricultural industry, boasting the world’s second-largest cattle herd and second-largest beef producer (Biscola et al. 2025). According to 2024 data from the Brazilian Institute of Geography and Statistics (IBGE, 2025), the Brazilian cattle herd exceeds 238 million head, with approximately 80% being either purebred or cross-bred Nellore (Nunes et al. 2024). In the country, these animals are raised in several states, including Pará, Mato Grosso, Mato Grosso do Sul, Minas Gerais, and Goiás (IBGE, 2025). The breed’s dominance is largely attributed to its remarkable adaptability to the tropical conditions of extensive pasture-based systems, which are prevalent in the country. Nellore cattle exhibit high tolerance to heat stress and parasites (Hansen 2004), and their pigmented skin, covered with short, dense white hair, enhances their productivity in challenging tropical environments, making them the foundation of beef production in Brazil and other tropical regions (Costa et al. 2018; Storti 2020).

In 2023, this robust production system enabled Brazil to export a record of 2.29 million tonnes of beef to 157 countries worldwide (ABIEC, 2024). Consequently, many breeding programs in Brazil have historically focused on improving Nellore productive and reproductive performance to meet the strong beef-market demand. For instance, recent studies in Polled Nellore cattle have reported moderate heritabilities for carcass traits, including 0.30 for longissimus muscle area, 0.13 for backfat thickness, and 0.15 for shear-force, indicating significant potential for genetic improvement in carcass-quality (Mamede et al. 2023). In the same population, heritability for scrotal circumference was 0.37, while heritability for age at first calving was 0.16, revealing that fertility and precocity can also be effectively enhanced through selection (Mamede et al. 2023). Similarly, research on Nellore cattle raised in the Pantanal region confirmed strong genetic control of early growth, with direct heritabilities along the growth curve ranging from 0.35 to 0.75 (e.g., 93.4 kg at 120 days, 180.4 kg at 205 days), confirming strong genetic control of early growth (Vaz et al. 2024). Further supporting this, a study by Forni et al. (2007) calculated the genetic trends for visual scores of conformation, precocity, and musculature at weaning, reporting values of 0.013; 0.022 and 0.018 respectively, representing an annual increase of 0.42; 0.67 and 0.60% per year in relation to the phenotypic average, respectively. Together, these findings illustrate the consistent genetic progress achievable in both growth and reproductive traits within the Nellore breed. Despite these advances, morphological defects persist as a significant challenge in Nellore production systems (Silva et al. 2023). A recent study performed by Campos et al. (2025a) analyzed over 180,000 phenotyped animals and reported the incidence rates for several structural defects. The most frequent conditions include feet and leg malformations (7.44%), followed by nose depigmentation (6.18%), hump abnormalities (5.59%), chamfer defects (5.05%), navel anomalies (4.84%), loin malformation (4.76%), and jaw misalignment (4.23%). These values suggest that a substantial proportion of animals in commercial herds are affected by at least one visible defect, which may compromise adaptability, animal welfare, and productivity. Although some breeding programs have included visual assessments on their evaluations, morphological defects are often simply treated as a criterion for disposal of animals (Silva et al. 2023; Carreño et al. 2019; Soares et al. 2024). This approach, however, fails to address the underlying genetic factors, prevalence, and effective methods for evaluation and management, treating them as aesthetic issues rather than significant factors that lead to premature culling and substantial economic losses for producers (Campos et al. 2025a).

Morphological traits and defects in cattle, such as body conformation, feet and leg structure, depigmentation, and craniofacial anomalies like chamfer and jaw defects, are critical determinants of performance, productivity, longevity, and welfare (Silva et al. 2023). For example, beef cattle with severe feet and leg malformations cannot move or graze efficiently, leading to economic losses. While breeders have traditionally used visual score systems to identify well conformed animals, the genetic architecture underlying these traits has not been deeply investigated (Carreño et al. 2019; Campos et al. 2025a).

Although numerous studies have investigated the genetic and genomic basis of morphological defects in Nellore cattle, the available evidence remains fragmented across traits, phenotyping systems, statistical models, and production environments. Differences in scoring methodologies, evaluator subjectivity, and analytical approaches hinder direct comparison of results and limit their integration into breeding programs. To date, no comprehensive review has consolidated the available genetic and genomic evidence on morphological defects in Nellore cattle within a unified methodological framework, highlighting the need for a structured synthesis of current knowledge gaps and methodological challenges. Accordingly, this review aims to evaluate and consolidate existing genetic and genomic evidence on morphological defects in cattle, with specific emphasis on the Nellore breed, in order to clarify current knowledge gaps, methodological limitations, and implications for genetic evaluation and breeding strategies.

## Materials and methods

Approval from the Welfare and Animal Use Committee was not required for this study as the data were obtained from previously published literature and no animals were directly involved in this research.

### Data sources and search strategy

This comprehensive review was performed through extensive searches of multiple databases to identify relevant literature. The literature search was performed across three major electronic databases: Web of Science, PubMed, and SciELO. The search targeted studies investigating the genetic basis of morphological defects (e.g., conformation, feet and legs malformations, navel abnormalities, pig-mentation defects, jaw and facial deviations), and reproductive defects (e.g., testicular hypoplasia, cryptorchidism) in purebred Nellore cattle (*Bos taurus indicus*). The targeted studies were the ones reporting genetic parameters (e.g., heritability, genetic correlations, variance components), and/or genetic evaluation models (e.g., linear, threshold, Bayesian, ssGBLUP). Searches were performed between May and September 2025. The complete search strings used for each database and the number of records retrieved per database are provided in Table 1. All retrieved records were imported into Covidence review software (www.covidence.org) for de-duplication, title and abstract screening, and general full-text eligibility assessment.

**Table 1:** Search strings used in each database query, by trait group

Trait group	Search string
Morphological traits	(“Nel$ore” OR “Bos indicus” OR “Zebu”) AND (“morphological defect” OR “structural defect” OR “skeletal abnormality” OR “jaw defect” OR “limb malformation” OR “umbilical hernia” OR “depigmentation” OR “conformation trait”) AND (“genome-wide association study” OR GWAS OR “genomic evaluation” OR “genetic parameters”)
Reproductive traits	(“Nel$ore” OR “Bos indicus” OR “Zebu”) AND (“reproductive trait” OR “fertility” OR “age at first calving” OR “sexual precocity” OR “scrotal circumference” OR “conception rate” OR “heifer pregnancy” OR “stayability”) AND (“genome-wide association study” OR GWAS OR “genomic selection” OR “genetic parameters”)
Methodology for measurements	(“Nel$ore” OR “Bos indicus” OR “Zebu”) AND (“morphological trait” OR “conformation trait” OR “structural soundness” OR “type trait” OR “linear type trait” OR “visual score” OR “linear score” OR “morphometric trait” OR “muscling” OR “feet and legs” OR “hump angle” OR “hump width” OR “hip height” OR “body depth” OR “jaw defect” OR “umbilical hernia” OR “navel” OR “pigmentation” OR “depigmentation” OR “locomotion”) AND (“heritability” OR “genetic correlation” OR “variance component” OR “genome-wide association study” OR GWAS OR “genomic evaluation” OR “genomic selection” OR “threshold model” OR “Bayesian inference” OR “ssGBLUP” OR “REML”) AND cattle OR “Bos indicus” OR Nellore) AND “visual assessment” AND (conformation OR morphological) AND (limitation OR bias) (cattle) AND (automated scoring OR phenomics) AND (body condition score OR conformation) AND (computer vision OR 3D imaging)

### Study selection and eligibility criteria

Titles and abstracts were screened independently by two reviewers, and disagreements were resolved through discussion or consultation with a third reviewer. Records considered potentially eligible underwent full-text assessment.

Studies were eligible for inclusion in this comprehensive review if they met the following criteria: (i) analyzed morphological traits recorded as visual or categorical scores (e.g., structure, precocity, muscling, navel/sheath development, pigmentation, feet and legs); (ii) applied genetic evaluation models such as linear mixed models via REML/BLUP, threshold models, Bayesian frameworks, multivariate models, or genomic approaches (e.g., ssGBLUP, wssGBLUP, GWAS); and (iii) reported heritability estimates, variance components, or genetic correlations for the traits of interest. Publications were excluded if they involved non-morphological traits, presented purely phenotypic analyses without genetic parameter estimation, included crossbred or taurine (*Bos taurus taurus*) populations, or represented duplicate datasets already included in other selected studies. No formal risk-of-bias or quantitative study-quality assessment tool was applied. Instead, methodological characteristics relevant to the interpretation of each study were examined descriptively, including the population and sample size, phenotype definition, scoring system, statistical model, and reporting of genetic or genomic results.

Genetic parameters reported in the eligible studies were extracted and summarized descriptively, without statistical pooling, due to the limited number of studies per trait and their heterogeneity in trait definitions, scoring systems, models, and populations. Only peer-reviewed articles, academic theses, and conference proceedings published between January 2000 and September 2025 in English or Portuguese were considered for inclusion. All genetic parameters extracted from the eligible studies were compiled into a summary table provided in the Online Resource 1. To facilitate the synthesis, studies were grouped according to trait category (e.g., feet and legs, navel, pigmentation, craniofacial defects) and methodological approach (genetic parameter estimation or genomic analysis).

## Results and discussion

### Study selection

The database searches retrieved 1,168 records, including 1,004 from Web of Science, 159 from PubMed, and 5 from SciELO. After the removal of 232 duplicates using Covidence, 936 unique records remained for title and abstract screening. Following full-text assessment, 89 articles met the inclusion criteria for at least one component of the review.

The studies included in the Nellore-specific synthesis differed in phenotype definition, scoring system, population size, and analytical approach. Table 2 summarizes the principal genetic and genomic findings by trait category, including the reported heritability estimates, genetic correlations, and major genomic regions or candidate genes. The complete study-level information is provided in Online Resource 1.

**Table 2:** Summary of genetic and genomic evidence for morphological traits and defects in Nellore cattle

Trait category	Phenotype or scoring system	Reported heritability	Main genetic associations	Main genomic findings
Facial defects	Chamfer and jaw defects evaluated indirectly through breed-quality or racial-aspect scores	Approximately 0.14–0.25 for composite breed-quality scores	An unfavorable correlation with scrotal circumference at 450 days was reported in one study (approximately − 0.25)	Direct genomic evidence for individual facial defects remains limited
Depigmentation	Binary classification based on presence or absence	0.12 on the observed scale and 0.54 on the liability scale	Genetic relationships with production and reproductive traits remain insufficiently characterized	Major signals on BTA22 near *MITF*, BTA6 near *KIT*, and BTA12 near *EDNRB*
Feet and leg defects	Binary classification and categorical scores ranging from undesirable to desirable	Approximately 0.18 for the binary phenotype and 0.39 for the categorical score	A favorable genetic association with yearling weight was reported when lower defect liability and higher weight were considered desirable	Polygenic architecture involving genes related to skeletal development, cartilage, muscle support, and metabolism, including *EXT1*, *SHOX*, *SCUBE3*, *ATG7*, *ITGA1*, and *PPARD*
Hump and loin defects	Binary classification of morphological deviation	Defect-specific estimates remain limited	Genetic correlations with other traits have not been clearly established	Significant regions were reported on BTA22 and BTA23 for hump defects and BTA25 for loin defects, with candidate genes including *IQSEC1*, *ACAD9*, *MYH11*, and *NDE1*
Navel defects	Visual scores generally recorded at weaning and yearling	Approximately 0.14–0.42, depending on age and analytical model	High genetic correlations across ages and sexes; associations with growth traits were generally weak or age-dependent	Major association on BTA5 involving an indicine-specific copy-number variant within *HMGA2*
Testicular defects	Binary classifications or ordered composite scores for hypoplasia, cryptorchidism, monorchidism, and asymmetry	Approximately 0.04–0.33, depending on phenotype and analytical scale	Associations with scrotal circumference ranged from negative to near zero across studies	Polygenic architecture involving steroidogenesis, oxidative protection, and cholesterol metabolism, including *STAR*, *KHDRBS3*, *GPX5*, and *INSIG1*
General visual scores	Structure, precocity, and muscling evaluated using 1–5 or 1–6 scales	Most estimates ranged from approximately 0.10 to 0.68	Genetic correlations among visual scores were generally positive and often high; associations with growth were usually favorable	Multiple genomic regions have been identified, supporting a polygenic architecture

### Breed characteristics and morphological defects in nellore cattle

**Fig. 1:**
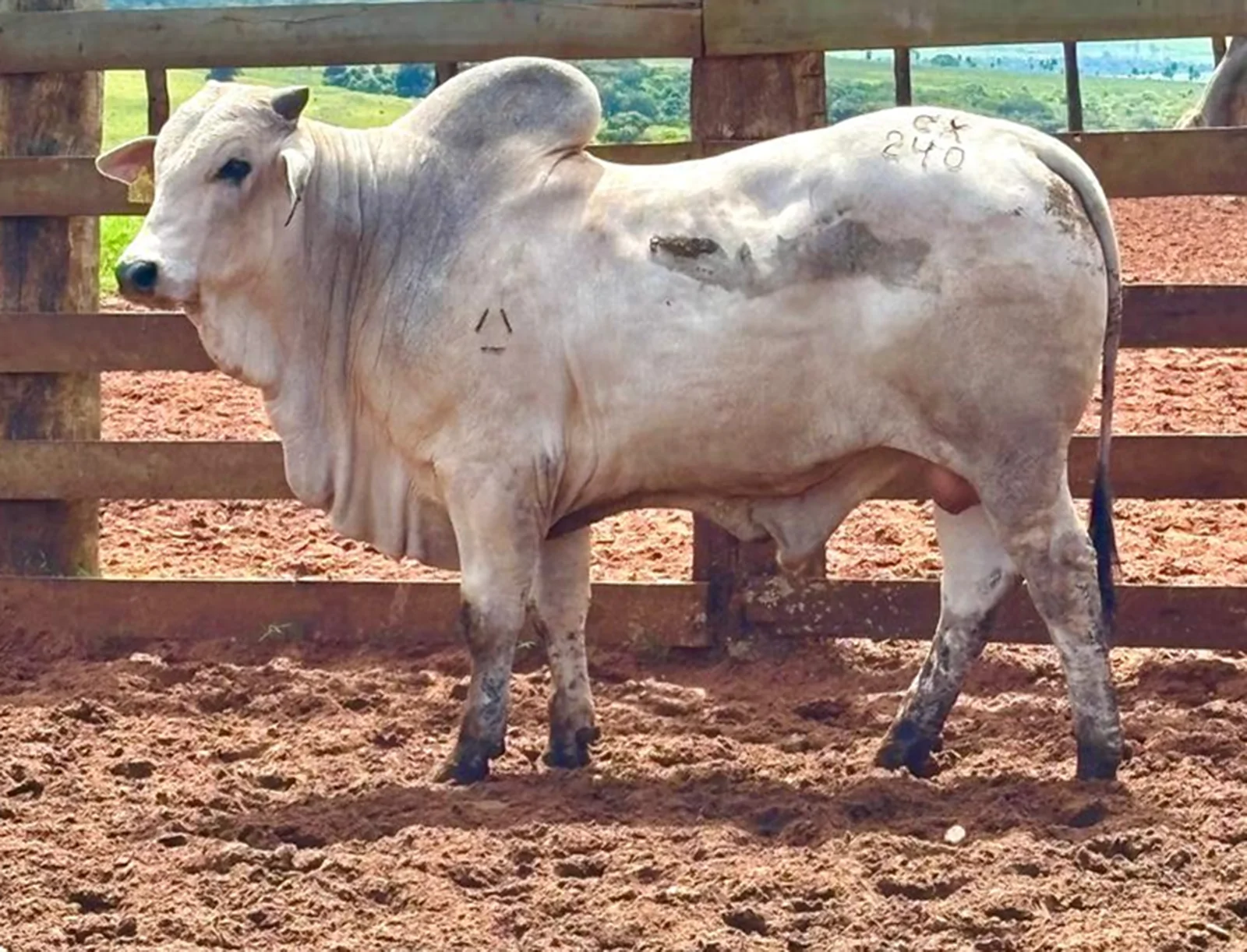
Example of a Nellore bull exhibiting normal conformation and no observable morphological defects. Photograph provided by Gensys^®^ and the DeltaGen^®^ breeding program

Nellore cattle are known for their adaptability to tropical environments, a characteristic intimately linked to their distinctive morphological features. The breed exhibits several adaptive traits including a prominent muscular hump positioned above the withers, a light gray to white coat overlying darkly pigmented skin, loose and elastic hide, relatively short ears, and long legs adapted to hot and marshy pasture conditions (Fig. 1). These morphological characteristics contribute not only to superior heat tolerance but also to remarkable resilience under extensive grazing systems typical of tropical beef production (Mason 1988). Because morphological characteristics are directly linked to animal function, health, and productivity in these production systems, they have become an essential focus in both traditional selection (e.g., cattle show, visual appraisals) and modern genetic improvement programs.

Nellore breeding programs and companies provide extensive technical information on their selection schemes and evaluation criteria through their official websites. To guide and standardize morphological evaluations, breed associations, breeding programs, and companies have established visual scoring systems that describe conformation and breed type (Brazilian Association of Zebu Breeders, ABCZ, www.abcz.org.br; Gensys^®^, www.gensys.com.br; BrasilcomZ^®^, www.brasilcomz.com; Geneplus, www.geneplus.com.br; Qualitas, www.qualitas.agr.br; and ANCP, www.ancp.org.br). These systems assign scores for multiple traits including muscularity, body structure, finishing precocity, breed characteristics, navel and sheath development, pigmentation, leg and feet structure, and sacrum alignment (Carreño et al. 2019). By quantifying visual appraisal into numerical scores, these systems enable objective recording of morphological variation, thereby facilitating genetic analyses and informed selection decisions. However, when functional morphologies deviate from desired breed standards, they are classified as morphological defects, i.e. undesirable conformation traits that may impair locomotion, reproductive efficiency, adaptation, or welfare. Such defects include feet and leg malformations (e.g., weak pasterns, sickle hocks, abnormal claws), pendulous navels, weak topline, chamfer deviations, falling hump, jaw defects, and depigmentation (Silva et al. 2023; Campos et al. 2025b). Understanding the full spectrum of morphological variation in Nellore cattle, from functional traits to undesirable defects, is essential for designing effective selection strategies that simultaneously improve adaptation, productive performance, and welfare in tropical beef production systems.

### Facial defects (chamfer and jaw)

**Fig. 2:**
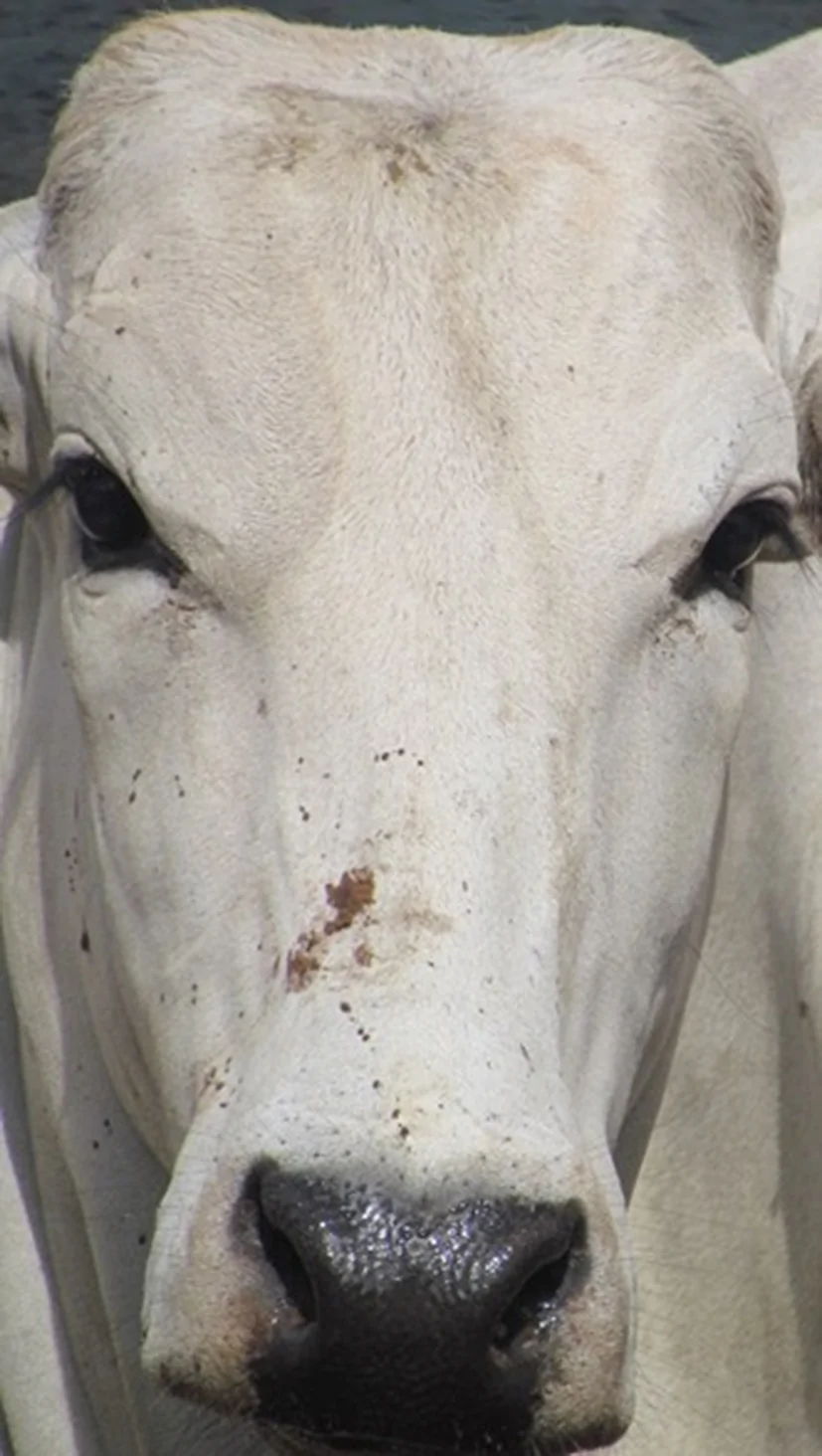
Nellore animal with chamfer deviation. Photograph provided by Gensys^®^ and the DeltaGen^®^ breeding program

Facial deviations in Nellore cattle are typically appraised within the composite Racial/Breed Quality score (R/BQ, also termed “Racial Aspects”), which serves as a comprehensive evaluation of conformity to breed standards (Soares et al. 2023). The scoring rubric explicitly considers chamfer deviation (commonly referred to as “crooked face”) (Fig. 2) and jaw malocclusion or articulation defects as disqualifying features that deviate from the breed standard. Animals free of these defects receive higher R/BQ scores, which are also used for breed registration and eligibility determinations by ABCZ (ABCZ, 2023). Across multiple studies performed in Nellore populations, direct heritability for R/BQ are consistently moderate, ranging from approximately 0.20 to 0.25 at typical evaluation ages (Soares et al. 2023; Faria et al. 2009; Koury Filho et al. 2010; Paterno et al. 2017; Regatieri et al. 2011). These heritabilities support the expectation of selection response via visual appraisal.

Notably, an unfavorable moderate genetic correlation between R/BQ and scrotal circumference at 450 days (SC450) has been reported (rg ≈ − 0.25 ± 0.17), indicating potential antagonism between conformation as captured by R/BQ and male fertility (Paterno et al. 2017). It is important to emphasize, however, that correlation does not imply causation, and findings such as these require further investigation before definitive conclusions can be drawn. Because chamfer and jaw defects are embedded within the composite R/BQ score, direct selection for higher R/BQ is expected to reduce facial defects indirectly. However, to avoid adverse correlated responses in reproductive traits, multiple-trait selection approaches are recommended (Soares et al. 2024). It is important to recognize that R/BQ is a composite conformation construct; therefore, estimates for R/BQ do not isolate facial defects per se but rather reflect broader racial type characteristics (Duitama et al. 2015; Faria et al. 2008). Between-study differences in scoring scales, evaluator training protocols, and age evaluation windows contribute to the heterogeneity in reported estimates. In a comparative biological context, malocclusions such as prognathism and brachygnathism occur at low prevalence in small ruminants and impair feeding efficiency, while in humans, mandibular prognathism is recognized as a polygenic trait with functional and masticatory consequences (Kalmari et al. 2023; Magalhães et al. 2008) These observations are contextual and should not be extrapolated to Nellore genetics beyond biological plausibility.

### Depigmentation

**Fig. 3:**
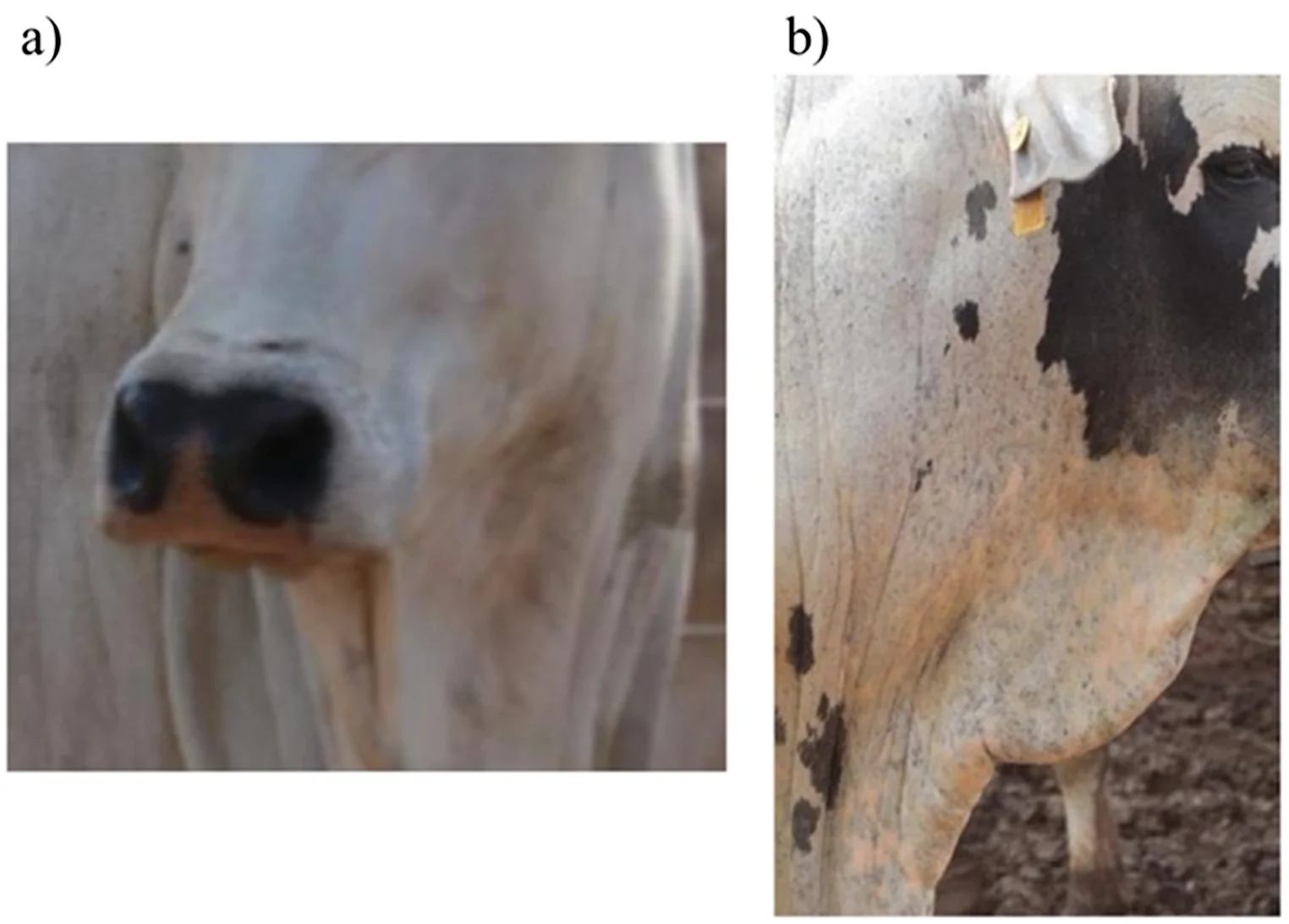
Nellore animal with depigmentation in the nose (**a**) and in the dewlap (**b**) Photograph provided by Gensys^®^ and the DeltaGen^®^ breeding program

Depigmentation in Nellore cattle is defined as the localized absence or loss of melanin, most frequently observed at the tail tip, mucous membranes, and as small patches distributed across the body (Fig. 3). Depigmentation is routinely analyzed as a binary phenotype (present/absent) in genetic evaluations (Campos et al. 2026). This condition is distinct from generalized white coat color, which reflects global melanogenesis failure despite the presence of melanocytes (Petersen et al. 2023). In tropical, pasture-based systems where Nellore are raised outdoors under intense solar radiation, the adaptive combination of reflective white hair over darkly pigmented skin provides essential thermal and ultraviolet (UV) protection (Costa et al. 2018; Gebremedhin et al. 2023; Silva et al. 2003). Loss of skin pigmentation compromises this critical defense mechanism, increasing susceptibility to sunburn, cutaneous lesions, and heat-related stress, with practical repercussions that include reduced market value, earlier culling, and in some breeding schemes, the loss of eligibility for breed certifications such as the Special Certificate of Identification and Production (CEIP) due to nonconformity with racial standards (Albuquerque et al. 2018; Carvalheiro et al. 2019). Increasing environmental heat loads and UV exposure associated with climate change may further aggravate these outcomes and could contribute to rising incidence of depigmentation over time (Chen et al. 2023; Colombi et al. 2024).

From a genetic standpoint, depigmentation exhibits substantial additive control and is expected to respond effectively to selection. The liability-scale heritability has been estimated at 0.54 (95% HPD 0.51–0.57), and approximately 0.12 on the observed (binary) scale, consistent with scale compression (Campos et al. 2026). Genome-wide association analyses in Nellore populations indicate a polygenic architecture with at least two major, partly independent biological routes affecting pigmentation biology. A primary signal on BTA22 encompasses the *MITF* locus and nearby genes such as *GNAI2*; conditioning on the strongest BTA22 promoter region signal eliminates the peak, consistent with a single major driver at this locus and with *MITF*’s well established role as regulator of melanogenesis (Campos et al. 2025b). An independent signal on BTA6 involves the *KIT* gene, which is required for melanocyte development, migration, and survival (Campos et al. 2025b; Vargas et al. 2022). Additional smaller effect loci have been reported on BTA12 near *EDNRB*, which also participates in melanocyte development and function (Campos et al. 2025b), while structural variant analyses have identified further candidate genes including *MAML2*, *UMAD1*, and *SAMD8* (Vargas et al. 2022). Functional enrichment analyses from primary genome scans highlight melanogenesis and UV response pathways; notably, the disappearance of pigmentation related terms after conditioning on *MITF* further underscores its central regulatory role (Campos et al. 2025b).

Interestingly, recent work by Campos et al. (*submitted*) shows that depigmentation expressed on the nose represents a distinct, tissue-specific phenotype, with a genetic architecture that does not overlap with the loci underlying whole-body skin depigmentation (Campos et al. 2026). Nasal depigmentation is evaluated on mucosal tissue, which differs developmentally and functionally from cutaneous skin, and genome scans identify major signals on BTA13 and BTA27 rather than the *MITF* and *KIT*-centered regions observed for generalized skin depigmentation (Campos et al. 2025a). This reinforces that pigmentation loss in different anatomical regions is governed by partially independent pathways and should not be treated as a single trait in genetic analyses (Campos et al., *submitted*).

From a breeding and management perspective, direct selection against the depigmentation phenotype should effectively reduce prevalence given its high heritability on the liability scale, while genomic evaluations incorporating markers from both the *MITF* and *KIT*-anchored regions are advisable to model non-redundant biological pathways (Campos et al. 2026; Carvalheiro et al. 2019). Regardless of genetic progress, management strategies to minimize UV exposure remain important for affected animals. Comparative studies across species consistently implicate conserved roles for *MITF* and *KIT* in white spotting and depigmentation phenotypes (Petersen et al. 2023; Dai et al. 2024; Flori et al. 2019), providing a broader biological context though not permitting direct inference of effect sizes in Nellore cattle.

### Feet and leg defects

Feet and legs malformation in Nellore cattle represents a functionally important binary defect (affected/unaffected) with an estimated incidence of approximately 5% in evaluated populations; with males reported to be more frequently affected than females (approximately 4.98% vs. 4.23%) (Silva et al. 2023; Vargas et al. 2018). Field assessment is typically performed around 550 days of age (yearling stage) and includes two related phenotypic records: (1) FL1, a binary indicator (0/1) for the presence or absence of feet and legs malformation; and (2) FL2, a categorical score ranging from 1 (undesirable) to 5 (desirable), used to qualify the top ~ 20% of candidates for breed certification (Vargas et al. 2018).

Animals coded as “1” (affected) on FL1 are generally excluded from FL2 evaluation (Vargas et al. 2018). Reported genetic parameters indicate moderate heritability for FL1 (posterior mean ≈ 0.18) and higher heritability for FL2 (≈ 0.39), reflecting meaningful potential for selection response and supporting the favorable genetic trends observed over time (Silva et al. 2023; Vargas et al. 2017). A moderate and favorable genetic correlation with yearling weight (YW) has been estimated at approximately − 0.23, when interpreted with lower feet and legs malformation liability and higher YW, both considered desirable (Vargas et al. 2017).

Genome-wide association studies performed in Nellore populations have revealed a complex genetic architecture for feet and legs malformation, implicating biological pathways related to skeletal development, muscle integrity, and metabolic regulation (Silva et al. 2023; Vargas et al. 2018). Prioritized candidate genes include *EXT1* (BTA14; involved in heparan‑sulfate–mediated regulation of chondrocyte proliferation and endochondral growth (Hilton et al. 2005), *SHOX* (BTA2; skeletal morphogenesis (Marchini et al. 2016), *SCUBE3* (BTA23; co‑receptor in *BMP2*/4 signaling and endochondral ossification (Lin et al. 2021), *ATG7* (BTA22; skeletal‑muscle development and autophagy; (Masiero et al. 2009), *ITGA1* (BTA20; extracellular‑matrix and cartilage structure (Black et al. 2023), and *PPARD* (BTA23; lipid‑metabolism and mechanical‑load adaptation (Magadum et al. 2018).

**Fig. 4:**
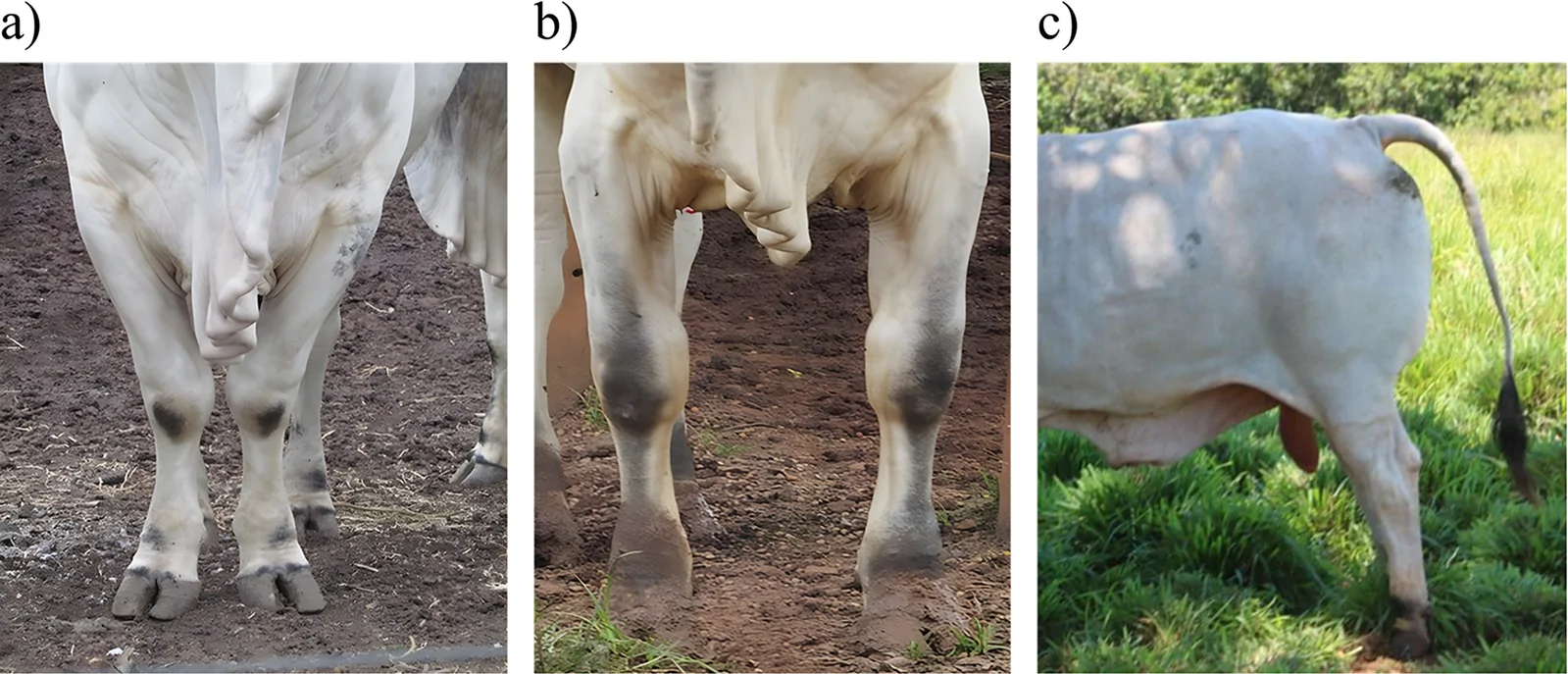
Feet and legs malformation observed in the Nellore cattle. (**a**) lateral view; (**b**) and (**c**) front view. Photograph provided by Gensys^®^ and the DeltaGen^®^ breeding program

Additional genomic regions influencing feet and legs conformation include *DLX2* (BTA2; osteogenic differentiation; (Zhang et al. 2019), *IL1B/IL1A* and *SLC20A1*/PiT1 (BTA11; phosphate transport in cartilage/bone (Couasnay et al. 2019), and *CTSL* (BTA8; bone resorption pathways (James et al. 2001). Collectively, these findings indicate that feet and legs malformation expression is influenced by a polygenic network linking endochondral ossification, cartilage and joint integrity, muscle support, and growth-related metabolism (Silva et al. 2023; Vargas et al. 2018). Similar conclusions have been drawn from studies where variation in hoof and limb conformation has been linked to skeletal and connective tissue genes (Abdalla et al. 2021), reinforcing the concept that feet and leg soundness is governed by conserved developmental pathways across cattle breeds (Sitz et al. 2023). These combined results highlight the importance of limb conformation as a key determinant of mobility, animal welfare, and productive longevity in pasture-based production systems. Figure 4 shows common malformation observed in feet and legs in Nellore cattle.

### Hump and loin defects

The hump of *Bos taurus indicus* cattle, such as Nellore, is a muscular structure positioned above the withers on the dorsal thoracic region, most developed in males and composed primarily of the cervical and thoracic portions of the rhomboideus muscle, with contributions from the trapezius, supraspinous ligament, and their fascial attachments (Utsunomiya et al. 2019). Although various adaptive and draught related hypotheses have been proposed to explain hump development, there is currently no robust evidence identifying a specific genomic region or major gene that determines hump development (Utsunomiya et al. 2019). The loin represents an economically important carcass region that reflects overall muscularity and commercial yield, commonly evaluated through the longissimus thoracis area, also known as the ribeye or loin eye area (REA/LMA), which is measured between the 12th and 13th ribs. A larger REA indicates greater edible meat portion and improved boning yield (Costa et al. 2023). Reported heritabilities for loin traits are typically moderate, such as LMA h² ≈ 0.30 in Polled Nellore populations (Mamede et al. 2023), suggesting substantial additive genetic control and potential for genetic improvement.

**Fig. 5:**
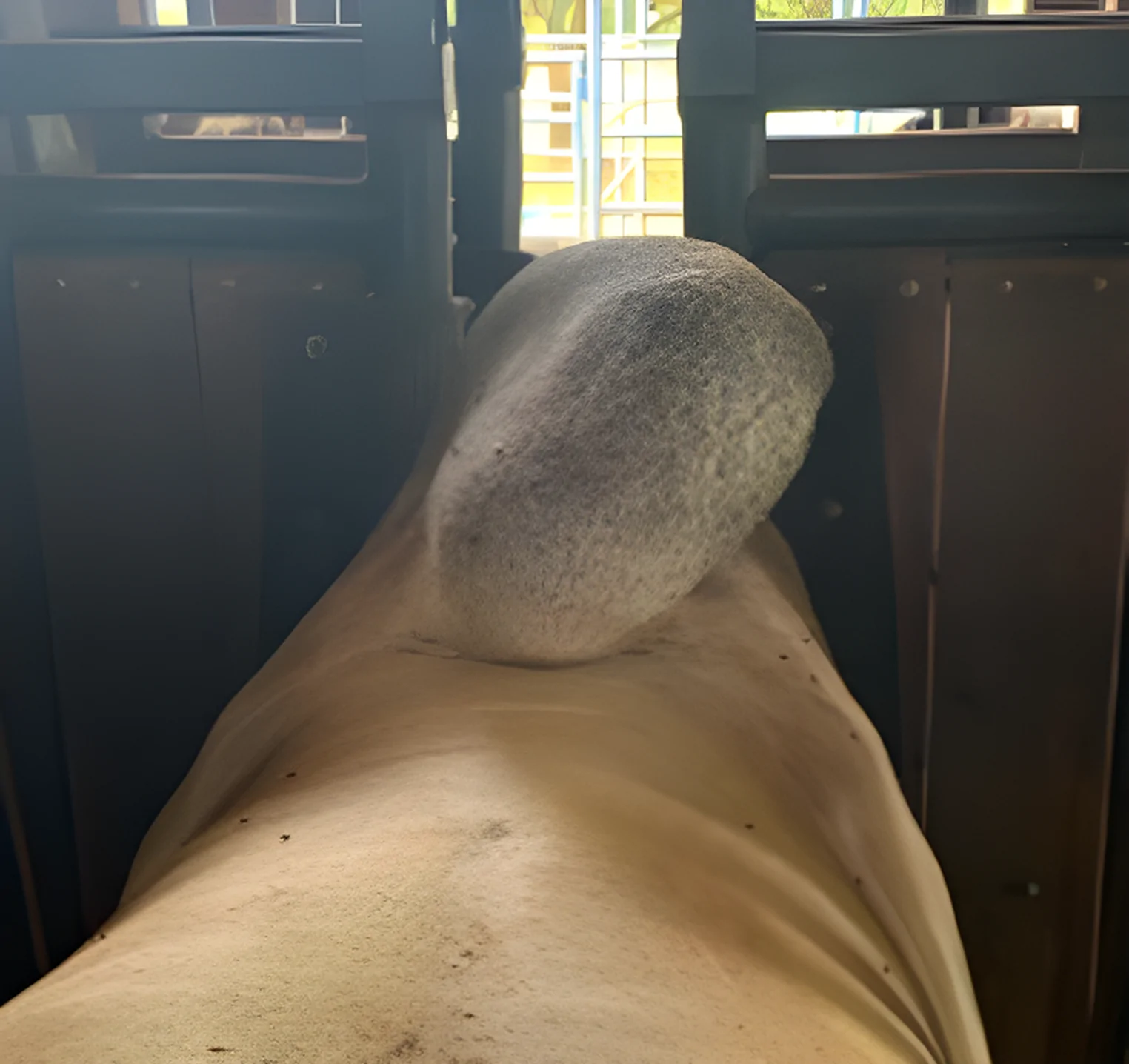
Fallen Hump. Photograph provided by Gensys^®^ and the DeltaGen^®^ breeding program

Although the hump and loin are normally described as anatomical and productive features characteristic of the breed, their deviations can also manifest as morphological defects, including fallen hump (Fig. 5) or loin asymmetry, which may negatively affect structural balance and market perception. Recent genomic analyses in Nellore cattle (Campos et al. 2025b) have identified significant SNPs for fallen hump on BTA22 and BTA23, and for loin deviation on BTA25, implicating genes related to signal transduction, fatty acid metabolism, and cytoskeletal organization (*IQSEC1*, *ACAD9*, *MYH11*, *NDE1*, *MARF1*, bta-mir-484, bta-mir-1246).

### Navel defects

Navel length is a routinely recorded and economically relevant trait in Nellore cattle raised under extensive pasture systems in Brazil. Excessively long or pendulous navels are particularly susceptible to mechanical abrasion, traumatic injury, and secondary bacterial infections, which increase veterinary treatment costs and may affect reproductive performance. In males, trauma or inflammation of the prepuce and sheath can progress to ulceration or constrictive fibrosis, potentially impairing penile exposure and natural service capability (Aguiar et al. 2018). Field evaluations are performed by trained technicians using a visual 1–5 scoring scale (where higher scores indicate longer or more pendulous structures), typically applied at weaning (WN) and again at yearling (YN). The breeding goal favors balanced, well directed navels and sheaths typically corresponding to intermediate scores of 2–3 (Faria et al. 2009).

Reported heritabilities for this trait range from h² = 0.22 ± 0.03 at weaning and h² = 0.42 ± 0.03 at yearling when estimated with threshold models, compared to 0.16 and 0.29 under linear models, respectively (Bignardi et al. 2011; Boligon et al. 2016). Genetic correlations between weaning and yearling scores, and between sexes, are strong and positive, indicating consistent phenotypic expression across ages and supporting the feasibility of joint selection strategies. The relationship between navel traits and growth appears to be age dependent. Selection for early growth tends to increase weaning navel length, as evidenced by positive genetic correlations with weaning weight and height, whereas yearling navel scores are largely independent of postweaning gain, enabling simultaneous improvement in both growth and navel conformation (Boligon et al. 2016).

At the molecular level Aguiar et al. (2018) performed a haplotype based GWAS for yearling navel, identifying a major signal on BTA5 (47.9–48.2 Mb). A ~ 6.2-kb copy-number gain within intron 3 of *HMGA2* was validated by qPCR and explained the association, an effect missed by conventional SNP-level analyses. The *HMGA2* variant likely influences navel morphology through regulation of the IGF axis (e.g., *IGF2*) and exhibits negative pleiotropy with precocity and muscling. Comparative analyses revealed that this CNV is indicine specific, reflecting morphological divergence between *Bos taurus indicus* and *Bos taurus taurus*. Earlier work by Koury Filho et al. (2003), using data from a single Nellore herd evaluated between 1972 and 1997, reported moderate heritability (h² ≈ 0.29) for yearling navel score and a high genetic correlation between males and females (rg ≈ 0.80). Genetic correlations between navel score and weaning weight (rg ≈ − 0.05) and between navel score and post weaning weight gain (rg ≈ 0.14) were weak, confirming the potential to select for shorter navels without adversely affecting growth performance.

### Testicular defects

Testicular defects represent important causes of reproductive inefficiency in beef cattle because they compromise sperm production, testicular thermoregulation, and overall breeding soundness. In Nellore bulls, the most frequently reported abnormalities include testicular hypoplasia, cryptorchidism, monorchidism, and asymmetry. These conditions differ in clinical presentation and genetic control, but all have practical consequences for fertility and herd management (Silva et al. 2023). Testicular hypoplasia is the best characterized defect and is defined by incomplete development of the seminiferous epithelium in the seminiferous tubules, which results in reduced sperm concentration, a higher frequency of morphological abnormalities, and in severe bilateral cases azoospermia (Silva et al. 2011). Hypoplastic testes are usually smaller and softer on palpation. The condition can be unilateral or bilateral, and partial forms may escape early detection because affected bulls often retain libido and some reproductive ability, which inadvertently favors unnoticed dissemination of the defect through natural service (Silva et al. 2011). In large scale Nellore evaluations that support the CEIP program, testicular hypoplasia is a frequent reason for bull disqualification, with an incidence close to 4–5% in yearling animals (Silva et al. 2023).

From a quantitative genetic perspective, testicular hypoplasia shows low to moderate heritability, for example h² about 0.16 with a 95% confidence interval of 0.13 to 0.22 in national Nellore datasets, which indicates meaningful additive variation and scope for selection response (Silva et al. 2023; Neves et al. 2019). Genetic associations with male fertility indicators are consistent with biological expectations. (Silva et al. 2011, 2023) reported moderately to highly heritable for scrotal circumference is in indicine bulls (0.42 to 0.46), and it is unfavorably correlated with testicular defects, such that selection for larger scrotal circumference tends to reduce the frequency of hypoplastic testes. Testicular volume is moderately heritable (approximately 0.31) and is very strongly genetically correlated with scrotal circumference at andrological evaluation (rg = 0.94), which supports scrotal circumference as a practical proxy for testicular development (Silva et al. 2011). Testicular shape also shows heritable variation (heritability near 0.20) and moderate positive genetic correlations with scrotal circumference (near 0.45 to 0.52) (Silva et al. 2011). However, selection toward more spherical testes in hot climates can impair thermoregulation and negatively affect sperm quality (Silva et al. 2011).

Monorchidism, characterized by the complete absence of one testicle and attributed to early testicular regression or undetected cryptorchidism, is also a disqualification criterion because it compromises fertility and complicates breeding soundness evaluation (Lamesch, 1994). Testicular asymmetry refers to visible or palpable differences in testicular size, shape, or firmness and may result from developmental imbalance, localized inflammation, trauma, or degenerative changes. Field studies of beef bulls have documented testicular asymmetry as a common reason for reproductive disqualification (Oliveira et al. 2020), and clinical reports describe scrotal asymmetry confirmed by palpation and morphometric analysis (Mosallam et al. 2024). Broader reviews of male genital pathologies in cattle also include asymmetry among the structural testicular disorders that can affect reproductive soundness (Butkiewicz et al. 2025). Moderate asymmetry may not severely impair fertility, but extreme differences are often associated with fibrosis, tubular degeneration, and reduced sperm output. Formal heritability estimates for testicular asymmetry in cattle are not yet available, though repeated occurrence in specific lineages suggests partial genetic control. For breeding programs, this phenotype should be systematically recorded together with scrotal circumference and semen quality traits to improve reproductive evaluations and enable future genetic analyses.

Because individual testicular defects often have low prevalence, a recent study proposed modeling them as a unified, ordered composite trait to improve the stability of genetic estimates (Campos et al., *submitted*). Using a five-level score that represented increasing severity across cryptorchidism, hypoplasia, monorchidism, and asymmetry, the study reported a heritability of approximately 0.33 on the liability scale, with a corresponding value of 0.07 on the observed scale. This pattern reflects the expected reduction in heritability due to scale compression and indicates that these abnormalities share partially overlapping genetic pathways despite their distinct clinical presentations.

At the molecular level, genomic studies in indicine cattle support a polygenic basis for testicular defects, implicating biological pathways related to steroidogenesis, lipid and cholesterol metabolism, oxidative protection, and spermatogenesis. Reported loci include *STAR* for acute steroid synthesis, *KHDRBS3* for post-transcriptional regulation in supporting cells, *GPX5* for protection of sperm membranes against lipid peroxidation, and *INSIG1* for regulation of *SREBP* and cholesterol homeostasis (Silva et al. 2023; Campos et al., *submitted*).

### Visual scoring systems for morphological evaluation

Visual scoring systems are a non-invasive, rapid, and low-cost alternative for identifying animals with superior productive conformation, making them a particularly valuable tool in large-scale beef cattle breeding programs (Faria et al. 2009) These systems provide an indirect assessment of carcass composition and finishing, and they also serve as a primary tool for detecting morphological defects, particularly those affecting structural soundness (Salgado 2019). Although protocols vary, most visual scoring methods aim to identify animals with a healthy, functional, and productive phenotype. The scoring systems often overlap with traits linked to physical soundness and, although originally developed to evaluate Muscling, Structure, and Precocity, these same features influence the anatomical development of skeletal and soft tissue structures associated with conformation related defects. As emphasized by Salgado (2019), critical defects such as foot and leg conformation and navel abnormalities are phenotypic characteristics capable of disqualifying an animal in a breeding program. These defects can be effectively evaluated using visual scores assigned by trained technicians.

One of the foundational scoring systems, the Morphological Evaluation System (MES) described by (Faria et al. 2009), focuses on Muscling, Structure, and Precocity. In this context, structure is based on body length and rib depth; precocity reflects fat deposition and limb proportion; and muscling is evaluated based on the distribution and volume of muscle across the body. Animals are scored from 1 to 6, with intermediate animals within each contemporary group serving as references (Bignardi et al. 2011; Faria et al. 2010; Koury Filho et al. 2009). Though not originally developed to identify defects, these traits are functionally correlated with skeletal conformation and fat deposition and can reveal hidden structural issues. Extending this concept, Sant’Anna et al. (2015) evaluated conformation, precocity, muscling, and navel traits at both weaning and yearling, using an average of three independent scorers to ensure consistency. Conformation was based on body size and muscle volume; precocity was associated with fat deposition and rib shape; and muscling involved assessments of the shoulder, loin, rump, and hindquarter. These visual scores were later correlated with both temperament and performance, highlighting how physical structure is interrelated with other important traits. Other authors, followed similar methodologies, typically using 1–5 scales and incorporating either evaluator consensus or average scores across multiple technicians, a strategy that reduces subjectivity and increases reproducibility of visual assessments (Forni et al. 2007; Regatieri et al. 2011; Boligon et al. 2016; Barrozo et al. 2015; Shiotsuki et al. 2009).

For evaluations where morphological defects are the primary focus, more detailed systems like EPMURAS and MERCOS are usually used. The EPMURAS system provides a granular assessment of Structure, Precocity, Muscularity, Navel, Breed characteristics, Posture, and Sexual characteristics, with most traits scored on a 1–5 scale (ABCZ, 2023; Lima et al. 2013). This system serves as a bridge between generalized conformation assessments and defect specific evaluations. Similarly, the MERCOS system is also frequently adopted in beef cattle breeding (Faria et al. 2008; Soares et al. 2023, 2024). It evaluates Muscling, Physical Structure, Conformation, Navel, and Sexual traits on a 1-to-5 scale (Lôbo 1996). According to (Faria et al. 2009), physical structure was assessed by evaluating hoof and ligament integrity, bone firmness, and skeletal width. Navel positioning and sacral development were also included, the latter scored as binary traits in some programs.

Visual assessment of navel and preputial traits has been formally incorporated into Nellore breeding programs as a practical approach for screening large herds under extensive conditions. An early standardized system for umbilicus score in Nellore, with a 1 to 5 scale ranging from very pendulous to very short navels, showed that this trait is heritable and that selection for improved navel conformation does not compromise weaning weight or post weaning gain (Koury Filho et al. 2003). Genetic evaluations based on large datasets later reported low to moderate heritabilities for umbilical scores and generally favorable or neutral genetic correlations with growth traits, confirming that these traits can respond to selection in Nellore populations (Faria et al. 2009; Bignardi et al. 2011). Studies that combined visual scores with ultrasound measures of body composition also demonstrated that visual evaluations capture meaningful variation in structure and fatness, reinforcing their usefulness as low-cost indicators for genetic improvement in beef cattle (Gordo et al. 2012). Importantly, visual scores have also been used to directly detect binary morphological defects. Studies by Vargas et al. (2018); (2017) and Silva et al. (2023) evaluated foot and leg abnormalities using binary scores (presence or absence), demonstrating the practical application of visual systems in screening structural soundness across large-scale herds.

### Methodological approaches for categorical morphological traits

Analyses of categorical morphological traits in Nellore cattle have evolved considerably over the past two decades, reflecting the growing need for models that accommodate the discrete and often skewed nature of visual scores. Early studies relied primarily on linear mixed models under the restricted maximum likelihood (REML) framework, which remain widely used due to their computational simplicity, efficiency, and ability to handle large datasets with complex pedigree structures. When trait distributions are relatively balanced and include several ordered classes, linear models generally provide heritability estimates and animal rankings comparable to those obtained with threshold models, supporting their continued use in large-scale evaluations (Faria et al. 2008, 2009; Resende et al. 2014).

However, the limitations of linear models become increasingly evident when dealing with traits expressed in few or highly unbalanced categories. In these cases, linear assumptions of normality are violated, therefore, linear models underestimate additive genetic variance, leading to biased heritability estimates (Faria et al. 2008; Bignardi et al. 2011; Hidalgo et al. 2024). To address these statistical limitations, researchers have increasingly adopted threshold models, which treat categorical phenotypes as discrete manifestations of an underlying continuous liability distribution (Falconer and Mackay 1996). Implemented primarily through Bayesian inference with Gibbs sampling algorithms, threshold models provide a more appropriate statistical representation for categorical and binary data and can be extended to multiple-trait frameworks that combine categorical and continuous variables within the same analysis (Campos et al. 2025a; Faria et al. 2008, 2009; Santana et al. 2013). In Nellore cattle, threshold analyses have yielded higher or more stable heritability estimates for traits such as structure, muscling, and navel development highlighting consistent genetic control across ages and sexes (Faria et al. 2009; Bignardi et al. 2011; Hidalgo et al. 2024). Across studies, heritability estimates for visual scores (e.g., structure, precocity, muscling, navel, pigmentation) are generally moderate to high, reinforcing their inclusion in breeding objectives. Because heritability is population and environment specific, direct comparisons among studies must be interpreted with caution, as variation in management conditions, population structure, trait definitions, scoring systems, and statistical models can substantially influence parameter estimates. Threshold models often produce slightly higher values for skewed traits, while linear REML tends to perform well for traits scored on 1–5 scales with approximately normal distributions. For instance, Paterno et al. (2017) reported heritabilities of 0.27–0.44 for weaning and yearling structure, precocity, and muscling using Bayesian two-trait models. Similarly, Faria et al. (2009) estimated h² = 0.44 (95% CI: 0.35–0.52) for yearling structure under a threshold model, while Bignardi et al. (2011) found h² = 0.42 and 0.36 for weaning and yearling navel development, respectively. Linear REML studies by Duitama et al. (2015) reported h² values of 0.24 (structure), 0.32 (precocity), and 0.27 (musculature) at yearling. Under genomic frameworks, Gordo et al. 2016 obtained similar heritabilities using both traditional BLUP and single step ssGBLUP (e.g., 0.23 for conformation, 0.27 for precocity, and 0.33 for muscling), demonstrating consistency across methodological approaches. For binary pigmentation defects, (Campos et al. 2026) estimated a heritability of 0.54 on the liability scale and 0.12 on the observed scale using a Bayesian single-step threshold model, illustrating how scale transformation affects the interpretation of genetic parameters. Reported additive genetic variances (σ_a_^2^) range from 0.28 to 0.77 (Gordo et al. 2012) for yearling conformation traits to 1.24 for structure (Faria et al. 2009), reflecting substantial genetic variability available for selection. High genetic correlations among EPM traits (structure, precocity, muscling) have been consistently observed, typically ranging from 0.76 to 0.94, indicating shared genetic control (Regatieri et al. 2011; Duitama et al. 2015; Koury Filho et al. 2009). Positive correlations with production traits, such as birth weight (rg = 0.58; (Faria et al. 2009) and hip height (rg = 0.21–0.25; (Santana et al. 2013) suggest that indirect selection for growth may improve visual scores simultaneously. Long term evaluations confirm favorable genetic trends, with annual genetic gains of approximately + 0.013 to + 0.022 score units for EPM traits and smaller but positive progress for navel (+ 0.002; (Silveira et al. 2021). Phenotypic means are typically around 3.8–3.9 for EPM traits and 2.9 for navel (Gordo et al. 2012), with most animals scoring between 3 and 4 (Faria et al. 2008)]. The integration of genomic information has further advanced the evaluation of morphological traits in recent years. The single-step genomic BLUP (ssGBLUP) method (Aguilar et al. 2010) merges pedigree and genomic relationships in a unified H matrix, enabling joint evaluation of genotyped and non-genotyped animals within the same analytical framework. This approach can be implemented under either linear or threshold models and extended to weighted ssGBLUP (wssGBLUP) to partition genomic variance into chromosome or window-specific components (Silva et al. 2023; Gordo et al. 2016). Genomic integration improves prediction accuracy and helps identify candidate genomic regions associated with morphological variation, thereby complementing classical quantitative analyses (Aguilar et al. 2010; Misztal et al. 2014). In addition to ssGBLUP, new methods tailored for binary traits and large genomic datasets have emerged, including the fastGWA-GLMM approach implemented in GCTA (Jiang et al. 2021). This method leverages sparse genomic relationship matrix (GRM) approximations and efficient algorithms to enable scalable genome-wide association studies (GWAS) under generalized linear mixed models. In Nellore cattle fastGWA-GLMM has recently been applied to categorical traits, offering results consistent with Bayesian threshold models while reducing computational demands (Campos et al. 2025b).

Complementing these developments, multivariate and dimension reduction methods have been applied to characterize the underlying structure of correlated morphological scores. Multiple-trait mixed models jointly analyze correlated traits to increase estimation accuracy and quantify indirect selection responses. Principal Component Analysis (PCA) has also proven useful to summarize correlated traits (e.g., conformation, precocity, and muscling) into composite indices or biotypes that describe overall body type and are suitable for use in selection indices (Vargas et al. 2018). In Nellore cattle, PCA of visual scores has explained up to 87% of the additive genetic variance in the first three components, supporting its application in multivariate genomic analyses and breeding programs.

### Limitations of visual assessments and novel approaches

Despite these methodological advances, visual scoring systems are subject to several important limitations that must be acknowledged. A primary concern is their inherent subjectivity (Duitama et al. 2015; Bezerra et al. 2024). Even trained evaluators may introduce bias, and grader specific tendencies can affect parameter estimates. For example, Bezerra et al. (2024) observed that one scorer consistently assigned lower values than others across all traits evaluated. Such inconsistencies across evaluators or breeding programs (Shiotsuki et al. 2009) contribute to heterogeneity in heritability estimates reported across studies and can reduce the accuracy of genetic evaluations when evaluator effects are not properly accounted for in statistical models.

Additionally, score distributions are often imbalanced, with few animals receiving extreme values (particularly the lowest scores), which limits statistical power and complicates the calibration of automated scoring systems based on image analysis or machine learning (Resende et al. 2014; Bezerra et al. 2024). For progressive traits such as depigmentation, misclassification can occur when animals are evaluated before the phenotype becomes visible, leading to underestimated prevalence and biased heritability estimates (Campos et al. 2026). Current recording systems also lack granularity regarding the anatomical location, spatial extent, or severity of defects, forcing complex and potentially heterogeneous phenotypes into simple binary categories that may obscure biological heterogeneity. Environmental heterogeneity is another source of noise: evaluations performed in diverse management groups or at suboptimal ages may yield inconsistent results, as morphological traits may not yet be fully developed (Faria et al. 2008)].

One practical mitigation strategy involves improving scoring protocols themselves. Evidence from cattle body condition scoring shows that a non-trivial portion of phenotypic variation arises from the evaluator, and that agreement improves when assessments from several trained scorers are combined (Paul et al. 2020). Using the mode or the average of independent scores can therefore produce more reliable phenotypes and reduce scorer-specific noise. In practice, consensus scoring also yields values that behave more consistently as continuous traits, facilitating the use of linear models in large-scale genetic evaluations. Modeling challenges further persist despite methodological improvements. Linear models are not theoretically suited for categorical data and may yield biased estimates (Duitama et al. 2015; Faria et al. 2008), especially for traits with few classes or skewed distributions (Bignardi et al. 2011). Conversely, Bayesian threshold models can face computational difficulties, including convergence issues in multi-trait or complex models (Faria et al. 2009), and require large datasets for stable inference. Small sample sizes or low genotype coverage can also inflate standard errors and reduce accuracy (Duitama et al. 2015; Gordo et al. 2016), limiting the practical utility of genomic predictions in smaller breeding programs.

### Economic and welfare implications of morphological defects

Morphological defects can lead to significant economic losses and welfare issues in beef cattle. Animals affected by foot and leg malformations, pendulous navels, depigmentation, or testicular abnormalities are often culled early, as these conditions compromise their functional capacity and overall efficiency. Such defects reduce productive lifespan and increase herd replacement rates, generating direct financial losses for producers (Silva et al. 2023; Campos et al. 2025a, 2025b; Boligon et al. 2016) also emphasized that although morphological defects occur at relatively low population frequencies, their impact is disproportionate, as they impair locomotion, feed efficiency, and reproductive performance, thereby increasing management costs and reducing herd productivity. In extensive tropical systems, where animals must travel long distances to access resources, structural integrity is essential for welfare and production efficiency.

Importantly, many of these morphological defects act as predisposing factors for clinical conditions, including chronic lameness, inflammatory joint disease, traumatic and infectious lesions, dermatological disorders, and reproductive pathologies, thereby linking structural abnormalities to disease development and compromised animal welfare. However, direct genetic evidence linking specific morphological defects or detailed body measurements to reproductive traits in Nellore cattle remains limited, with most available studies reporting indirect associations or evaluating these traits independently.

According to Silva et al. (2023), the prevalence of feet and leg malformations in Nellore cattle is approximately 5%, with males more frequently affected (4.98% vs. 4.23% in females). These defects impair locomotion, predispose animals to inflammatory and degenerative joint conditions, reduce grazing ability, and often lead to premature slaughter. From a welfare perspective, Vargas et al. (2018) reported that inadequate limb conformation is associated with pain, inflammation, and higher susceptibility to joint injuries, highlighting the functional and ethical implications of these conditions. Similarly, pendulous navel and sheath defects increase the risk of trauma and infections, leading to higher veterinary costs (Aguiar et al. 2018). In the Aguiar et al. (2018) study, a haplotype-based GWAS identified variants related to navel length associated with infertility in rodents, suggesting that similar developmental pathways could negatively affect bull fertility in cattle, causing reproductive and economic losses. Depigmentation exposes animals to higher risks of sunburn and skin lesions, reducing both welfare and market value (Campos et al 2026). Testicular hypoplasia, which affects about 4.6% of bulls, results in disqualification for breeding and exclusion from CEIP certification programs, leading to genetic and financial losses for breeders (Neves et al. 2019).

### New technologies applied to genomic studies

Research areas related to animal breeding have benefited from the development of statistical models tailored to the practical needs of producers. The development and application of methodologies such as selection indices, mixed model equations, and Best Linear Unbiased Prediction (BLUP) have enabled progress in studies that directly influence livestock production systems (Chafai et al. 2023). These methodologies are well established and validated, with widely recognized strengths and limitations. They are robust methodologies that provide consistent results, particularly for traits that are easily measured or highly heritable; however, they still produce considerable uncertainty when addressing non-additive effects such as dominance, epistasis, and pleiotropy (Chafai et al. 2023). As animal breeding moves into the era of omics, exploring this new frontier requires the adoption of advanced analytical approaches, with artificial intelligence (AI) emerging as a powerful tool for extracting meaningful patterns from large and complex datasets, including images.

Artificial intelligence, which constitutes a computational simulation of human reasoning, encompasses a range of techniques, including machine learning (ML) and deep learning (DL). Machine learning involves training algorithms to generate predictions or make decisions from data, with performance improving as the system gains experience. Different problems require distinct ML models, such as linear regression, decision trees, random forests, and neural networks (Chafai et al. 2023; IBM, 2024). Deep learning, a subset of ML, employs deep neural networks that more closely approximate human decision making. A widely used DL method is the Convolutional Neural Network (CNN), which can autonomously extract key features from raw data through end-to-end learning, making it highly efficient for analyzing complex datasets like images (Goyache et al. 2001).

The application of AI in animal science dates back to early attempts to automate visual assessments. In 2001, Goyache et al. (2001). proposed using AI to “clone” the behavior of trained evaluators for cattle classification, though they faced challenges with image acquisition and inconsistent trait definitions. More recently, ML has been widely applied for assessing morphometric traits. A review by Yang et al. (2025) highlighted the relevance of computer vision and ML for feature extraction, pattern recognition, and classification in production animals. For example, (Tarr et al. 2025) developed an AI model to classify Limousin bulls, finding that its performance was comparable to traditional regression models, confirming the suitability of ML-based approaches for such tasks.

These advanced analytical methods rely on high quality input data, often derived from imaging technologies that can replace or supplement human evaluators. Studies have validated the use of digital photography and 3D imaging for assessing conformation traits. Vlček et al. (2017) compared two methods for evaluating hoof conformation: ruler measurements versus digital photographs in 120 Slovak Spotted dairy cows. Six parameters were evaluated, and five showed no significant differences between methods. The authors concluded that both approaches are effective for hoof assessment. The same group conducted a similar study involving 127 Holstein cows to evaluate the presence or absence of hoof disorders and concluded that photometric assessment can be effectively implemented in dairy systems. Similarly, Nisbet et al. (2022) examined the accuracy of 3D imaging equipment for morphometric measurements of Angus cattle using eight suspended 3D cameras capturing 60 potential traits. The study concluded that optimal performance requires controlled environments free from interfering with external factors.

Do et al. (2024) conducted a study focusing on preprocessing techniques for 3D images and the extraction of body traits in cattle. The study sought to reduce costs and human error in phenotyping morphometric traits of beef cattle. A total of 1,114 Charolais animals were scanned using a 3D system consisting of 10 synchronized cameras. Preprocessing included noise removal, alignment in a coordinate system, and defining a repeatable partial volume. The methodology successfully processed 90% of the images. Although promising, these technologies still face significant challenges, such as high cost, reduced accuracy in complex environments, difficulties balancing real time performance with hardware constraints, and limited applicability across species. These barriers currently limit wide spread adoption among different producer profiles (Yang et al. 2025; Summerfield et al. 2023).

Image processing is commonly performed using machine learning, as demonstrated by O’Mahony et al. (2023) in their study on estimating body condition score (BCS) with camera-based imaging. The authors found that 2D technologies can outperform 3D systems by leveraging broader datasets and pretrained models. They also highlighted concerns regarding the reliability of manually collected data: training algorithms using data from a single evaluator on one farm may compromise prediction accuracy on other farms. O’Mahony et al. (2023) investigated the integration of ensemble modeling with depth camera data, identifying which camera configurations provided superior results and comparing their computational approach with other predictive methods for body score estimation in Holstein cows. The three-camera configuration improved accuracy relative to one or two cameras, although gains did not always offset the increased cost.

The integration of artificial intelligence with new phenotyping technologies is a rapidly expanding field. Studies consistently report positive outcomes, indicating that these technologies can be successfully applied in the production of animal derived foods. Automated prediction systems for economically important traits can reduce human subjectivity and enhance estimation accuracy. Although no studies were identified for Nellore cattle, the positive results across other breeds suggest strong potential for detecting morphological defects in Nellore populations.

## Conclusions

This comprehensive review synthesized two decades of research on the genetic basis of morphological defects in Nellore cattle, demonstrating that these traits are biologically relevant, heritable, and suitable for inclusion in genetic improvement programs. Across traits such as depigmentation, feet and leg malformations, navel defects, craniofacial deviations, and testicular abnormalities, heritability estimates were generally low but still indicating additive genetic variation. Genomic studies revealed mostly polygenic architectures, with major loci identified for specific defects, including *MITF* and *KIT* for pigmentation and *HMGA2* for navel development.

Methodologically, both linear and threshold models have been applied, but threshold and Bayesian approaches offer more appropriate inference for categorical and unbalanced traits. Visual scoring systems remain the foundation of largescale phenotyping; however, their effectiveness is limited by evaluator subjectivity, imbalanced score distributions, and lack of anatomical detail. Evidence from consensus scoring demonstrates that averaging evaluations from multiple technicians can enhance phenotype reliability and reduce scorer-generated noise. Emerging imaging and machine-learning technologies offer promising avenues for automated, scalable phenotyping, though applications in Nellore cattle are still scarce.

Although this review focuses on Nellore cattle, many of the genetic pathways and methodological challenges discussed (e.g., categorical phenotyping, threshold modeling, evaluator subjectivity) are shared across cattle breeds and are relevant to genetic evaluation programs globally. Overall, the findings support the feasibility and importance of incorporating morphological defects into routine genetic evaluations to improve adaptability, longevity, and welfare in tropical production systems. Progress will depend on standardized scoring protocols, improved phenotyping strategies, and expanded genomic analyses that capture both major effect loci and polygenic background variation. Strengthening these areas will enable more accurate selection decisions and contribute to sustainable genetic progress in Nellore herds.

## Supplementary Information

Below is the link to the electronic supplementary material.

Supplementary Material 1

## Data Availability

No new data were created or analyzed in this study. All data used come from previously published studies, which are cited in the reference list.
