# Supplementary material for: Genetic and genomic insights into morphological defects in nellore cattle: a comprehensive review
Source: Trop Anim Health Prod. 2026 Oct 18;58(8):619. doi: 10.1007/s11250-026-05433-8 (PMC13677639; doi:10.1007/s11250-026-05433-8)
Supplement: Supplementary file 1 [file 11250_2026_5433_MOESM1_ESM.pdf]

Tropical Animal Health and Production

Review paper

Genetic and Genomic Insights into Morphological Defects in Nellore Cattle: A Comprehensive Review

Milena Aparecida Ferreira Campos[12], Hinayah Rojas de Oliveira[2*], Louise Sarmento Martins de Oliveira[1], Henrique Alberto Mulim[2], Eduarda da Silva Oliveira[3], Gregorio M. F. de Camargo[1] and Raphael Bermal Costa[1]

Federal University of Bahia, School of Veterinary Medicine and Animal Science, Salvador, Bahia, Brazil;

Purdue University, Department of Animal Sciences, West Lafayette, Indiana, USA;

São Paulo State University, School of Agriculture and Veterinary Science, Jaboticabal, SP, Brazil;

Milena Aparecida Ferreira Campos: 0000-0003-2198-235X

Hinayah Rojas de Oliveira: 0000-0002-0355-8902

Louise Sarmento Martins de Oliveira: 0000-0003-3094-3870

Henrique Alberto Mulim: 0000-0001-8798-8899

Eduarda da Silva Oliveira: 0009-0000-9855-2708

Gregorio M. F. de Camargo: 0000-0002-8736-5576

Raphael Bermal Costa: 0000-0002-6463-7436

*Correspondence: hinayah@purdue.edu;

S1. Summary of genetic parameters extracted from the eligible studies.

Reference	Sample size (phenotyped / genotyped)	Traits studied	Method	Heritability (h²)	Genetic correlations	Genomic regions / candidate genes
**Vargas et al. (2018)**	104,725 (FL1: 96,836; FL2: 14,708) — 1,435 (HD + 50K imputed to HD)	Feet and leg conformation (FL1 binary, FL2 categorical 1–5)	GWAS using weighted single-step GBLUP (wssGBLUP)	FL1: 0.16 ± 0.03; FL2: 0.44 ± 0.10	—	FL1: Chr 1, 2, 6, 7, 8, 10, 14; FL2: Chr 1, 7, 10, 11, 18, 20, 22, 28, 29 — DLX1, DLX2 (skeletal/limb development); IL1A, IL1B (cartilage integrity, inflammation); SLC20A1/PiT1 (bone physiology); CTSL (joint/bone disease)
**Silva et al. (2023)**	295,031 (FLM binary) — 12,537 (imputed HD, 461,057 SNPs after QC)	Feet and legs malformation (FLM, binary 0/1), yearling weight (YW)	Linear-threshold model (Bayesian, THRGIBBS1F90); GWAS using weighted single-step GBLUP (WssGBLUP); Gene prioritization with GUILDify + ToppGene	FLM: 0.186 (95% CI: 0.146–0.225); YW: 0.472	YW–FLM: −0.23 ± 0.01	12 windows >1% variance: BTA2, 3, 5, 6, 14, 18, 20, 22, 23 (2), 27, 29 (16.37% variance explained) — ATG7 (skeletal muscle autophagy); EXT1 (bone growth, chondrocytes); ITGA1 (lipid metabolism, intramuscular fat); PPARD (skeletal muscle fatty acid oxidation); SCUBE3 (BMP signaling, bone growth); SHOX (skeletal atavism, limb deformities)
**Carreño et al. (2019)**	2,873 animals with phenotypes (conformation, precocity, muscling, visual scores at weaning) — ~2,873 genotyped with Illumina HD (777K SNPs → ~310K SNPs after QC)	Visual scores at weaning: conformation (C), precocity (P), muscling (M), scored 1–5	GWAS using Bayesian methods: BayesC and Bayesian LASSO	C: 0.44, P: 0.43, M: 0.42 (threshold model)	—	Significant windows on BTA1, 3, 5, 7, 14, 15, 16, 19, 20, 23 — 18 candidate genes identified, including: IGFBP6, WFIKKN2, C1QTNF3, PPP1R39, ESRRG, STK3, MAPK11, MAPK12, NDUFA13, FBXL17, LPAR2, TMEM9, SORL1, ACSF2, CREG1, POU2F1, GSTA3
**Rajawat et al. (2024)**	Morphometric traits (qualitative: coat color, horn type, ear length; quantitative: body weight, chest girth, rump width, etc.) — Gir (20, ddRAD), Sahiwal (20, ddRAD), Tharparkar (20, 50K SNP chip), Kankrej (10, 50K SNP chip), Nelore (24, 50K SNP chip)	Morphometric/conformation traits	Selection signature analyses (Tajima's D, CLR via SweeD, iHS, ROH)	Not directly estimated (focus on selection signatures, not variance components)	—	Multiple breed-specific selective sweeps across autosomes — Kankrej: BCL6, SST, DPP6, INSIG1, SLC35D2, CYLD; Tharparkar: LPL, ATP6V1B2, CDC14B, MCM6, MYOG, CLCN2, NAV2, UNC5D; Nelore: HPSE2, PLAG1, CHCHD7, SDR16C5/6, FAM110B; Sahiwal: PCSK1, PRKD1, CSGALNACT1, TRIM68, SLC16A7, GHSR, ABL2, RUNX1T1; Gir: GNA14, GNAQ, HPCAL1, DOCK1, CCDC117, GRIP1
**Santana Jr. et al. (2013)**	121,086 animals (HH, PRE, MUS, BONE, HP); pedigree of 167,854 animals across 12 farms — Not genotyped (pedigree-based study)	Hip height (HH), finishing precocity score (PRE), muscling score (MUS), bone score (BONE), heifer pregnancy at 14 months (HP)	Bayesian multi-trait linear-threshold model (THRGIBBS1F90)	HH: 0.36; PRE: 0.26; MUS: 0.26; BONE: 0.26; HP: 0.50	PRE–MUS: 0.99; PRE–BONE: 0.85; MUS–BONE: 0.85; PRE–HP: 0.25; MUS–HP: 0.24; HH–HP: −0.06; BONE–HP: 0.03	—
**Faria et al. (2009)**	~1,000–3,800 animals per age class (8, 15, 22 months, and adult cows), total pedigree: 52,361 animals — Not genotyped (pedigree-	Visual scores: musculature, physical structure, racial aspects, conformation, navel (ôfalo), sacrum	Bayesian threshold animal model (MTGSAM) with Gibbs sampling (500k–1.1M cycles)	8 months: MUS 0.32, STR 0.60, RAC 0.22, CON 0.34, NAV 0.57, SAC 0.08; 15 months: MUS 0.38, STR 0.14, RAC 0.21, CON 0.07, NAV 0.36,	Positive correlations among MUS, STR, and CON (moderate to high); RAC and SAC showed lower consistency across ages; low correlations between traits	—

	based study)			SAC 0.46; 22 months: MUS 0.21, STR 0.27, RAC 0.20, CON 0.29, NAV 0.38, SAC 0.26; Adults: MUS 0.01, STR 0.07, RAC 0.18, CON 0.11, NAV 0.36, SAC 0.23	across distant ages (e.g., weaning vs maturity)	
**Faria et al. (2009)**	Visual scores: ~1,894 (15 mo), ~3,864 (22 mo); Carcass traits: ~2,000 animals (2002–2005) — Not genotyped (pedigree-based)	Visual scores (musculature, structure, conformation, sacrum) at 15 and 22 mo; Carcass traits by ultrasound: longissimus muscle area (AOL), backfat thickness (EG), rump fat thickness (EGP8), hip height (ALTG)	Bivariate linear-threshold Bayesian models (MTGSAM with Gibbs sampling, 500k–700k cycles)	Not explicitly reported for all traits in this paper (heritabilities in companion papers)	AOL correlations: MUS15 0.94, STR15 0.93, CON15 0.91; MUS22 0.87, STR22 0.91, CON22 0.85. Backfat correlations: MUS15 0.41, STR15 0.05, CON15 0.14; MUS22 0.45, STR22 0.45, CON22 0.50. Rump fat correlations: MUS15 0.16, STR15 –0.24, CON15 –0.03, SAC15 0.38; MUS22 0.48, STR22 0.02, CON22 0.44, SAC22 0.34. Hip height correlations: MUS15 0.08, STR15 0.77, CON15 0.62; MUS22 –0.08, STR22 0.71, CON22 0.63.	—
**Vargas et al. (2018)**	355,524 animals (1990–2010)	Growth: birth-to-weaning gain (BWG), weaning-to-yearling gain (WYG); Visual scores: conformation (WC, YC), finishing precocity (WP, YP), muscling (WM, YM); Reproduction: scrotal circumference (SC)	Multi-trait mixed linear animal model (Wombat); PCA (eigen-decomposition of additive genetic covariance matrix, AT)	BWG 0.17, WC 0.17, WP 0.21, WM 0.20, WYG 0.18, YC 0.34, YP 0.34, YM 0.34, SC 0.46	Positive correlations, ranging from 0.15 (WC–SC) to 0.96 (WP–WM); across ages 0.15–0.86	—
**Soares et al. (2023)**	4,175 animals scored for visual traits; 3,075 with reproductive records	Visual scores: muscularity (M), physical structure (PS), racial aspects (R), conformation (C), navel (N), sacrum (SAC); Reproductive traits: SC365, SC450, stayability (STAY), probability of precocious calving (PPC30), age at first calving (AFC)	Threshold-linear Bayesian model (THRGIBBS1F90)	Visual scores: 0.15–0.28 (PS highest); Reproductive traits: SC365 0.50, SC450 0.53, STAY 0.38, PPC30 0.54, AFC 0.10	Notable estimates: SC365–C 0.25; SC450–R –0.25; STAY–C 0.36, STAY–SAC 0.66; PPC30–SAC –0.55, PPC30–M –0.22; AFC–N –0.24; other correlations low (–0.20 to 0.15)	—
**Aguiar et al. (2018)**	2,016 animals used in GWAS (deregressed EBV-based pseudo-phenotypes for navel length at yearling) — 2,231 HD-genotyped Nellore (953 bulls + 1,278 cows) available; 503,088 SNPs used after QC; WGS for 21 Nellore	Navel length at yearling (visual score; analyzed via deregressed EBVs); secondary checks on nine routinely recorded traits (e.g., precocity, muscling)	Haplotype-based GWAS with LOCO in GCTA (haplotypes coded as pseudo-SNPs); tested 2,048,168 six-marker haplotypes; follow-up with WGS coverage, read-pair orientation, and qPCR validation	— (not estimated in this study)	Not a correlation study; HMGA2-tag haplotype showed weak but significant associations with visual precocity and muscling in regression on dEBVs	BTA5: 47.9–48.2 Mb peak ($p = 1.01 \times 10^{-9}$); CNV at 48,074,233–48,080,443 (intron 3 of HMGA2) — HMGA2 (intron 3 CNV); pathway context with IGF2 and PLAG1

	bulls; additional WGS from 5 African B. indicus and 4 European B. taurus breeds for origin analysis					
**Bignardi et al. (2011)**	UD 90,383; UDM 47,506; UDF 42,619; US 45,057; USM 17,666; USF 27,181; PD 128,700; PS 44,227 — — (pedigree-based; pedigree size 168,256)	Navel score at weaning (UD; sexes combined and by sex), navel score at yearling (US; sexes combined and by sex), weaning weight (PD), yearling weight (PS). Scores 1–5 (higher = longer/more pendulous).	Animal model, uni- and bi-trait; REML (MTDFREML). Random: direct additive; maternal additive (UD, UDM, UDF, PD, PS); maternal permanent environment (UD, UDM, UDF, PD); residual. Fixed: contemporary group; covariates for dam age and animal age.	Uni-trait h²: UD 0.14±0.01; UDM 0.18±0.02; UDF 0.15±0.01; US 0.26±0.01; USM 0.32±0.03; USF 0.27±0.02; PD 0.29±0.01; PS 0.27±0.02. Bi-trait h² (with PD/PS): UD 0.15; US 0.27; PD 0.29; PS 0.45.	Between sexes: UDM–UDF 0.85; USM–USF 0.75. Across ages: UD–US 0.93. Navel vs growth: UD–PD 0.33; UD–PS 0.17; US–PD 0.12; US–PS 0.06 (all positive, low–moderate).	—
**Boligon et al. (2016)**	Not reported in abstract (yearling breed quality; navel at weaning & yearling) — — (pedigree-based; no genotypes used)	Breed quality score at yearling (BQ); navel development at weaning (WN) and yearling (YN); growth traits: BWG (birth–weaning gain), WH (weaning height), WYG (weaning–yearling gain), YH (yearling height)	Linear vs threshold animal models; compared prediction ability and rank correlations of sire EBVs	BQ: 0.14 ± 0.01 (linear), 0.15 ± 0.02 (threshold). WN: 0.16 ± 0.01 (linear), 0.22 ± 0.03 (threshold). YN: 0.29 ± 0.01 (linear), 0.42 ± 0.03 (threshold).	Rank r between sires' EBVs from linear vs threshold: 0.53–0.98. BQ with BWG 0.27 ± 0.02; BQ with WYG 0.25 ± 0.02. WN with BWG 0.63 ± 0.02 and WH 0.53 ± 0.02; WN with WYG −0.06 ± 0.02 and YH 0.26 ± 0.02. Weak rg for YN with growth.	—
**Neves et al. (2019)**	46,256 records kept after editing (47,563 initial; TH incidence 4.61%) — 265 sires (8 on 50K, 257 on HD; imputed to HD; 500,689 SNPs)	Testicular hypoplasia (TH), binary at 18 mo	Single-trait threshold animal model (Bayesian) to estimate EBVs; BayesC GWAS using de-regressed EBVs (pseudo-phenotypes); windows of 5 adjacent SNPs	Posterior mean h² = 0.16 (95% HPD 0.08–0.23)	Genetic correlations of TH with selection traits ranged −0.53 to 0.04; SC vs TH = −0.53; with selection index at weaning IW = −0.32; combined index IFINAL = −0.36	Top windows on BTA1, 3, 4, 5, 9, 22, 23, 25; together explain 22.69% of additive variance; strongest window on BTA4: 117.95–117.96 Mb (~9.88%) — Examples: INSIG1, PAXIP1 (steroid metabolism, cell survival/DNA damage response); additional positional candidates listed in Table 2
**Silva et al. (2011)**	SC at 18 mo (SC18): 50,809; Andrological SC (SCAND): 17,211; Testicular volume & shape: 15,313 — — (pedigree-based; no genotypes)	Scrotal circumference at 18 mo (SC18); SC during andrological exam (SCAND); testicular volume; testicular shape; minor defects; major defects; total defects; satisfactory andrological evaluation	Multi-trait animal model with Bayesian Gibbs sampling (Gibbs2F90); fixed effects: contemporary group; covariate: age; random: direct additive	SC18: 0.42; SCAND: 0.43; Volume: 0.31; Shape: 0.20; Minor defects: 0.04; Major defects: 0.16; Total defects: 0.15; Satisfactory androl.: 0.10	SC18 with: SCAND 0.84, Shape 0.75, Volume 0.44, Minor −0.23, Major −0.16, Total −0.24, Satisfactory 0.56; SCAND–Volume 0.94; SCAND–Shape 0.52; Volume–Shape 0.52	—
**Silva et al. (2023)**	207,195 yearling records; TH incidence 4.6% — 17,326 animals after QC; 461,057 SNPs (HD ref panel; medium-density imputed to HD)	Testicular hypoplasia (TH, 0/1 at ~18 mo); correlated analysis with scrotal circumference (SC)	Linear–threshold animal model (Bayesian, THRGIBBS1F90) in two-trait (SC–TH) for EBVs & rg; WssGBLUP for GWAS with non-overlapping 20-SNP windows; QC: HWE p<1e−5, call rate>0.90, MAF≥0.02; imputation with FImpute; H-matrix per Aguilar et al.	TH h² = 0.168 (95% CI 0.130–0.215); SC h² = 0.462 (0.441–0.482)	rg(SC, TH) = −0.0445 ± 0.0227 (near zero, slightly unfavorable)	12 windows >1% (sum 18.04%): BTA1 (2), 7, 9, 11 (2), 13, 14, 20, 24, 27, 28; top window BTA14: 6.12–6.17 Mb (2.28%) — Prioritized/positional: STAR (BTA27); GPX5 (BTA1); ERLIN2 (BTA27); KHDRBS3 ~219 kb from top BTA14 window
**Koury Filho et al. (2003)**	EU (navel score, 18 mo): 11,310; EUM (males):	Navel visual score at ~18 mo (1 = more pendulous/long, 5	Two-trait animal model; REML (MTDFREML); fixed	EU 0.29; EUM 0.30; EUF 0.30; PD 0.31; GP345 0.14	EU–PD −0.05; EU–GP345 0.14; male–female navel rg =	—

	5,089; EUF (females): 6,221; PD (weaning wt): 13,191; GP345 (weaning→yearling gain, 345 d): 7,874 — — (pedigree-based; no genotypes)	= shorter/adhered); growth: PD and GP345	CG + age covariates; additive direct (all traits) + maternal additive/permanent env. for PD		0.80	
**Abreu Silva et al. (2018)**	Database of ~480,000 animals used for (co)variance estimation — — (pedigree-based; no genotypes)	Mature weight (MW) of cows; weaning weight (WWd); post-weaning gain 345 d (PWG); scrotal circumference (SC); muscling score (MUSC); probability of pregnancy at 14 mo (PP14)	Animal model, REML (multi-trait); estimated h² for WWd, PWG, SC, MUSC, PP14 and genetic correlations of MW with those traits	WWd 0.22 ± 0.01; PWG 0.19 ± 0.01; SC 0.50 ± 0.01; MUSC 0.22 ± 0.01; PP14 0.50 ± 0.01 (MW h² not stated in the abstract/records consulted) (AGRIS)	MW with: WWd 0.55 ± 0.04; PWG 0.68 ± 0.03; SC 0.07 ± 0.02; MUSC 0.46 ± 0.01; PP14 −0.03 (low, negative). Selection on WWd/PWG/MUSC is expected to increase MW; little to no genetic change in fertility from selection on MW. (AGRIS)	—
**Barrozo et al. (2015)**	C: 58,947; P: 58,611; M: 54,935 (long–yearling, ≈450 d; after edits) — — (pedigree-based; none)	Visual scores at long–yearling: Conformation (C), Precocity (P), Muscling (M) (1–5 scale)	Unitrait animal model, REML (MTDFREML); CG and direct additive as random; tested fixed covariates (age of dam at calving, long–yearling age, long–yearling weight) as linear/quadratic; compared models M1–M4	Across models: C: 0.13–0.36; P: 0.32–0.36; M: 0.35–0.38 (all indicating response to selection)	Not estimated (unitrait analyses only; model-comparison focus)	—
**Boligon, Mercadante & Albuquerque (2011)**	36,757 females with visual scores; mature cow weight recorded (subset for MW) — — (pedigree-based; no genotypes)	Visual scores at weaning (≈205 d) and yearling (≈550 d): Conformation (C), Finishing precocity (P), Muscling (M); Mature cow weight (MW)	Bivariate animal models, Bayesian (Gibbs): threshold for scores + linear for MW	SCW 0.16 ± 0.02; SFW 0.20 ± 0.02; SMW 0.19 ± 0.02; SCY 0.24 ± 0.02; SFY 0.31 ± 0.02; SMY 0.32 ± 0.02; MW 0.46 ± 0.04. (AGRIS)	rg(MW with scores): 0.27 ± 0.06 to 0.36 ± 0.04 (positive, moderate). Selection on higher C/P/M—especially at yearling—increases MW. (AGRIS)	—
**Bonin et al. (2015)**	12,447 individuals; visual scores + rump measures + ultrasound traits — — (pedigree-based; no genotypes)	Visual body scores: Conformation (CONF), Precocity (PREC), Muscularity (MUSC); rump width (RW), rump length (RL), frame; ultrasound: ribeye area (REA), backfat thickness (BFT), rump fat thickness (RFT)	Animal model (REML); estimated genetic parameters, trends, and progress	CONF 0.23±0.02; PREC 0.33±0.03; MUSC 0.31±0.02; RW 0.25±0.05; RL 0.16±0.04; Frame 0.31±0.03; REA 0.38±0.03; BFT 0.44±0.04; RFT 0.47±0.04.	CONF–RW 0.74±0.09; CONF–RL 0.82±0.08; CONF–Frame 0.83±0.04; REA–MUSC 0.39±0.05; REA–BFT −0.09±0.04; REA–RFT −0.03±0.04; PREC–BFT 0.36±0.07; PREC–RFT 0.29±0.07; Frame–BFT −0.02±0.03; Frame–RFT 0.05±0.05.	—
**Bezerra et al. (2024)**	23 elite bulls; 232 images (116 profile + 116 dorsum); concurrent records for BW, PET performance (FW, ADG, DMI, RFI), SC, LEA	Visual structure score (EPMURAS) by 3 graders; image-derived measures (profile: CH, WH, RH, SL, SVH, SA; dorsum: CW, DL, DA); BW and productivity metrics	Overhead camera setup; ImageJ feature extraction; Pearson correlations (scores, image metrics, BW, productivity); k-means clustering on top-correlated features; ANOVA/Tukey	—	—	—
**Boligon et al. (2017)**	Weaning scores: WC 541,788; WP 541,949; WM 541,981; WWG 543,148; Yearling scores:	Visual scores (Conformation, Precocity, Muscling) at weaning and yearling; Birth→weaning	Bayesian animal models: THRGIBBS1F90 (threshold for scores) & GIBBS1F90 (linear for gains & SC); tested	Direct h² (±SD): WC 0.20±0.01; WP 0.17±0.01; WM 0.15±0.01; WWG 0.33±0.02; YC 0.32±0.01;	High within-weaning rg: 0.70–0.95; WWG–WC 0.97. Same score across ages: WC 0.90; WP 0.88; WM 0.84.	—

	YC 316,931; YP 316,957; YM 316,957; SC 128,866; YWG 293,912	gain (WWG); Weaning→yearling gain (YWG); Scrotal circumference (SC)	maternal genetic/permanent env. at weaning; selection indices defined for weaning & yearling; chains 500k iters (burn-in 200k)	YP 0.28±0.02; YM 0.31±0.02; SC 0.37±0.02; YWG 0.18±0.01. Maternal h² (weaning): WC 0.10; WP 0.09; WM 0.08; WWG 0.06	With SC: weaning traits 0.35–0.61, yearling traits 0.36–0.53. Yearling: YP–YM 0.78; YWG with YC/YP/YM 0.41–0.43	
**Cancian et al. (2014)**	86 yearling males (42 non-castrated; 44 castrated); postweaning (~15 mo) and finishing (~21 mo) measures	Visual scores: Conformation (C), Precocity (P), Muscling (M); Ultrasound: UBFT, URFT, UREA; Feed efficiency: G:F, RFI; Carcass: HCW, DP, RPY	Pearson phenotypic correlations; ANOVA for phase×sex effects; ultrasound by UGC-certified tech; feedlot trial with individual feed intake	—	—	—
**Silveira et al. (2021)**	Growth/height/visual scores in large national dataset (approx. records): BW ~206k; WW ~432k; YW ~299k; HH ~190k; visual scores (CS, PS, MS) ~301k each; NS ~299k; BQ ~190k; carcass at yearling: LMA ~6,478; BF ~5,777	Growth: BW, WW, YW; Visual scores at yearling: breed quality (BQ), conformation (CS), muscling (MS), navel (NS), finishing precocity (PS); hip height (HH); carcass: backfat (BF), longissimus area (LMA)	Bayesian animal models (threshold for scores; linear for weights/HH/carcass); validation models for direct/maternal BW & WW; genetic trend analysis	Direct h² (means): BW 0.30±0.01; WW 0.24±0.01; YW 0.44±0.01; HH 0.37±0.01; CS/PS/MS 0.31–0.37 (moderate); Maternal h²: BW 0.03±0.01; WW 0.08±0.01; Carcass h² low (small n). (Scribd)	Growth with CS/PS/MS/HH: rg 0.20–0.94 (positive); with NS: 0.11–0.20 (low); with BQ: 0.14–0.37 (favorable). Growth↔carcass: null–moderate. (PubMed)	—
**Carreño et al. (2019)**	2,873 weaning visual-score records — 2,873; Illumina HD → ~309,865 SNPs after QC	Weaning visual scores: Conformation (C), Precocity (P), Muscling (M)	Bayesian GWAS (BayesC, Bayesian LASSO); dependent vars: phenotypes (threshold) and de-regressed EBVs (linear); 1-Mb windows	C: 0.44; P: 0.43; M: 0.42	—	BTA 1, 3, 5, 7, 14, 15, 16, 19, 20, 23 (several shared across C/P/M) — IGFBP6, WFIKKN2, C1QTNF3, PPP1R39, ESRRG, STK3, MAPK11/12, NDUFA13, FBXL17, LPAR2, TMEM9, SORL1, ACSF2, CREG1, POU2F1, GSTA3, TBC1D5
**Duitama et al. (2015)**	E/P/M: 7,256; GPD: 7,253; P18: 7,251; PE: 2,026	Visual scores at ~18 mo (Estructure, Precocity, Muscling; 1–6 scale); GPD (daily gain during test), P18 (weight at 18 mo), PE (scrotal circumference)	REML, linear animal model (WOMBAT). Fixed: contemporary group, covariates (final age/weight as applicable); random: direct additive only; pedigree 20,364 (1,949 sires; 11,157 dams); PX-EM + AI convergence, logL tol 1e−9. Contemporary groups defined within each trial after a 70-day adaptation; trials ran 224 days on Brachiaria forage (mineral + water ad lib.).	E 0.30 ± 0.04; P 0.37 ± 0.04; M 0.32 ± 0.04; GPD 0.26 ± 0.04; P18 0.37 ± 0.04; PE 0.44 ± 0.08.	Among VS: E–P 0.77 ± 0.04; E–M 0.76 ± 0.04; P–M 0.95 ± 0.02. VS↔GPD: E 0.45 ± 0.09; P 0.50 ± 0.08; M 0.46 ± 0.08. VS↔P18: E 0.83 ± 0.04; P 0.81 ± 0.04; M 0.80 ± 0.04. VS↔PE: E −0.04 ± 0.14; P −0.05 ± 0.13; M −0.03 ± 0.14. Also GPD–P18 0.78 ± 0.04; GPD–PE −0.21 ± 0.14; P18–PE −0.08 ± 0.13.	—
**Faria et al. (2009)**	Visual scores: 15 mo — M/E/C 1,894, SAC 1,666; 22 mo — M/E/C 3,864, SAC 3,434. Carcass/height: AOL 2,035; EG 2,022; EGP8 1,990; Hip height 811	Visual (MERCOS): Musculature (M), Structure (E), Conformation (C) (1–5), Sacrum (SAC) (binary) at 15 & 22 mo; Ultrasound carcass: Longissimus area (AOL), backfat (EG), rump fat (EGP8); Hip height (ALTG)	Bayesian bivariate linear–threshold animal model (MTGSAM/Gibbs), categorical ↔ continuous; CG as random for VS; long chains (500k–700k); pedigree 52,361; convergence checked with GIBANAL	— (focus of paper is genetic correlations; h² values not detailed numerically)	AOL with VS: at 15 mo M 0.94, E 0.93, C 0.91; at 22 mo M 0.87, E 0.91, C 0.85. EG with VS: 15 mo M 0.41, E 0.05, C 0.14; 22 mo M 0.45, E 0.45, C 0.50. EGP8 with VS/SAC: 15 mo M 0.16, E −0.24, C −0.03, SAC 0.38; 22 mo M 0.48, E 0.02,	—

					C 0.44, SAC 0.34. Hip height with VS: 15 mo M 0.08, E 0.77, C 0.62; 22 mo M −0.08, E 0.71, C 0.63.	
**Faria et al. (2009)**	8 mo: MERCOS n=1,041; SAC n=909. 15 mo: MERCOS n=1,894; SAC n=1,666. 22 mo: MERCOS n=3,864; SAC n=3,434. Adult cows: MERCOS n=3,156; SAC n=2,697.	Visual scores (MERCOS): Musculature (M), Structure (E), Racial aspects (R), Conformation (C), Ônfalo/navel (Ô), Sacrum (SAC); (scores 1–5; SAC binary).	Bayesian animal models with threshold for categorical traits; bi-trait linear–threshold when paired with continuous P210; MTGSAM/Gibbs: total 500k–1.1M cycles; burn-in 100k, thinning 1,000; contemporaneous group as random for VS; maternal additive included at weaning.	h^2 (posterior means) — 8 mo: M 0.32, E 0.60, R 0.22, C 0.34, Ô 0.57, SAC 0.08. 15 mo: M 0.38, E 0.14, R 0.21, C 0.07, Ô 0.36, SAC 0.46. 22 mo: M 0.21, E 0.27, R 0.20, C 0.29, Ô 0.38, SAC 0.26. Adult: M 0.01, E 0.07, R 0.18, C 0.11, Ô 0.36, SAC 0.23.	Within-age (genetic): 8 mo: M–E 0.40, M–C 0.61, E–C −0.07. 15 mo: M–E 0.23, M–C 0.47, E–C 0.38. 22 mo: M–E 0.18, M–C 0.75, E–C 0.56. Across-age: correlations between 8 vs 22 mo for the same trait are near zero; moderate when ages are adjacent (8–15 or 15–22 mo).	—
**Faria et al. (2008)**	3,864 animals scored for visual traits (22 mo); 19,093 for weight at 210 d (P210)	Visual scores (1–5): Musculature (M), Structure (E), Conformation (C); continuous: P210	Bayesian animal models: uni- & bi-trait threshold vs linear (MTGSAM, Gibbs); CG treated as random for VS; sex fixed; for P210, CG fixed & dam age class fixed; pedigree 52,361	Univariate (threshold vs linear): M 0.14 vs 0.12; E 0.15 vs 0.14; C 0.10 vs 0.11. Bivariate (P210 with VS), linear–threshold: P210 0.19–0.25; M 0.21; E 0.27; C 0.29–0.31. Bivariate linear–linear: P210 0.19–0.25; M 0.19–0.20; E 0.28–0.29; C 0.33.	P210↔VS (linear–threshold): 0.62 (M), 0.94 (E), 0.89 (C); linear–linear: 0.61, 0.86, 0.85. VS↔VS (threshold–threshold): M–E 0.18–0.20, M–C 0.75–0.76, E–C 0.56–0.60.	—
**Faria et al. (2009)**	Weaning: 5,407; Yearling: 2,649	Visual scores (MES/SAM): Structure (S), Precocity (P), Musculature (M) (1–6 scale)	Bayesian two-trait threshold animal model (MTGSAM/Gibbs); CG treated as random; sex & dam age fixed; long chains with burn-in/thinning; W120 (120-d weight) included for checks	Weaning: S 0.68, P 0.65, M 0.62. Yearling: S 0.44, P 0.38, M 0.32.	Genetic r with W120: Weaning: S 0.92, P 0.94, M 0.99. Yearling: S 0.94, P 0.62, M 0.72. Rank (EPD) correlations across ages: S 0.78, P 0.61, M 0.66.	—
**Freitas et al. (2020)**	BW (12 mo): 231,416; Visual scores S, P, M: 21,057 (8–14 mo); AFC: 15,750 (22–36 mo)	Principal component (PC) from visual scores (Structure, Precocity, Muscling); Body weight at 12 mo (BW); Age at first calving (AFC)	REML (REMLF90) mixed model; fixed: contemporary group + cow age at calving (lin+quad); random: direct additive, maternal permanent env., residual; PCA for PC (1st component)	BW: 0.51; PC: 0.30; AFC: 0.20	Additive genetic r: BW–PC: 0.48; BW–AFC: −0.31; PC–AFC: −0.55	—
**Gordo et al. (2012)**	W120 46,157; W550 9,765; LMA 13,285; BF 13,061; RF 12,811; BS 3,484; FP 3,484; MS 3,483; SN 3,303	Growth: W120 (120-d BW), W550 (BW at ultrasound). Ultrasound: LMA (LM area), BF (12–13th rib backfat), RF (rump fat). Visual scores: BS (body structure), FP (finishing precocity), MS (muscling), SN (sheath & navel)	REML (WOMBAT), multi-trait animal model. Fixed: contemporary group (year/season of birth, sex, mgmt), covariates: animal age ± quadratic; dam age (for some traits). Random: direct additive; maternal effects tested (kept for W120 in model testing; negligible for others in multitrait).	W550 0.37 ± 0.03; LMA 0.33 ± 0.03; BF 0.24 ± 0.02; RF 0.28 ± 0.03; BS 0.24 ± 0.04; FP 0.38 ± 0.05; MS 0.29 ± 0.05; SN 0.38 ± 0.06.	Highlights from 9-trait matrix: W120–W550 0.84; W120 with BS 0.87, LMA 0.49, FP 0.37, MS 0.47, SN −0.01. W550 with BS 0.91, LMA 0.55, FP 0.41, MS 0.55, SN 0.03. VS–LMA: 0.37–0.44. FP/MS–BF/RF: moderate positive (e.g., BF with FP 0.33, MS 0.40). SN shows low r with BS and body-composition traits.	—
**Koetz Júnior et**	Mature weight (cows):	Growth: BW, WG	Bivariate animal model	h^2 (direct): MW (mature)	—	—

**al. (2017)**	40,340; BW: 241,471; WG: 339,963; WC/WP/WM (weaning scores): 285,632 / 285,640 / 285,638; YG: 181,426; YC/YP/YM (yearling scores): 186,293 / 186,292 / 186,285; Yearling weight (YW): 137,562	(birth→weaning gain), YG (weaning→yearling gain), YW (yearling weight), MW (mature cow weight). Visual scores (1–5): WC/WP/WM at weaning; YC/YP/YM at yearling.	(DMU) to estimate (co)variances for MW with each trait; REML; fixed effects for CGs and covariates by trait; maternal additive & permanent effects modeled for growth traits (not for MW). Genetic trends from regression of mean EBV (×2) by birth year (SAS REG).	0.45; BW 0.34; WG 0.18; YG 0.18; YW 0.27; WC 0.18; WP 0.20; WM 0.17; YC 0.25; YP 0.27; YM 0.27.		
**Koury Filho, Albuquerque, Alencar, Forni & Chiquitelli Neto (2006)**	Yearling visual scores (males): 1,367; Hind height (HH): 2,128; Yearling weight (YW): 2,255	Visual scores at yearling (1–6 within CG): E body structure, P finishing precocity, M muscling; plus HH and YW	REML (MTDFREML) one- & two-trait animal models; fixed: CG (herd, sex, birth year/season, mgmt; 68 groups), age (linear + quadratic); random: direct additive	E 0.24 ± 0.09; P 0.63 ± 0.12; M 0.48 ± 0.11; HH 0.37 ± 0.08; YW 0.29 ± 0.07	E–P 0.49 ± 0.17; E–M 0.63 ± 0.15; P–M 0.90 ± 0.05; E–HH 0.57; P–HH −0.29; M–HH −0.33; E–YW 0.83; P–YW 0.42; M–YW 0.50	—
**Koury Filho et al. (2009)**	Visual scores at ~550 d: 1,367; Hip height (HH): 2,128; Weight at 550 d (W550): 2,255	Visual scores at yearling (~550 d): Structure (E), Precocity (P), Muscling (M) (1–6 within CG); plus W550 and HH	REML (MTDFREML) one- & two-trait animal models; fixed: contemporary group & age (lin+quad); random: direct additive	E 0.24 ± 0.09; P 0.63 ± 0.12; M 0.48 ± 0.11; HH 0.37 ± 0.08; W550 0.29 ± 0.07	Among scores (genetic): E–P 0.49, E–M 0.63, P–M 0.90. Scores with W550: E 0.83, P 0.42, M 0.50. Scores with HH: E 0.57, P −0.29, M −0.33	—
**Koury Filho et al. (2010)**	Weaning (≈184 d): 27,523 (Conformation, Precocity, Musculature, Weight). Yearling (~517 d): 21,746 (Conformation, Precocity, Musculature, Weight)	Visual scores (1–5 within CG): Conformation (C), Finishing precocity (P), Musculature (M) at weaning & yearling; body weights at the same ages	REML (REMLF90) animal models; fixed: CG, calf age (lin+quad), dam-age class; random: direct additive; for weaning traits also maternal additive and maternal permanent environment; multi-trait (bi/tri) analyses	Weaning: C 0.13, P 0.25, M 0.23, W 0.23; maternal h² (weaning): C 0.05, P 0.04, M 0.06, W 0.11. Yearling: C 0.24, P 0.32, M 0.27, W 0.37	Among scores (genetic): Weaning: C–P 0.67, C–M 0.61, P–M 0.95. Yearling: C–P 0.75, C–M 0.71, P–M 0.95. Weights with scores: Weaning W–C 0.97; W–P 0.67; W–M 0.62. Yearling W–C 0.83; W–P 0.59; W–M 0.58	—
**Shiotsuki et al. (2009)**	Pr16: 30,407; HH: 7,226; YW: 46,901; C: 46,919; F: 46,917; M: 46,909 (yearling; scores 1–5 within CG by 3 examiners)	Heifer pregnancy at 16 mo (Pr16), hip height (HH), yearling weight (YW), and yearling visual scores: conformation (C), finishing (F), muscling (M)	Bivariate animal models: linear for HH, YW, C, F, M; threshold for Pr16; Bayesian Gibbs (MTGS), flat priors; 400k–600k rounds, 5k burn-in; convergence via GIBANAL	h² (mean ± SD): Pr16 0.49 ± 0.03; HH 0.30 ± 0.02; YW 0.34 ± 0.01; C 0.15 ± 0.01; F 0.21 ± 0.02; M 0.23 ± 0.02 (90% CIs reported)	rg (Pr16 with): HH −0.34 ± 0.07, YW 0.08 ± 0.03, C 0.05 ± 0.04, F 0.18 ± 0.03, M 0.16 ± 0.03; residual r also reported (≈0.27–0.31)	—
**Paterno et al. (2017)**	W210: 33,242; W450: 26,259; Weaning VS (BSW/FPW/MSW): 23,075; Yearling VS (BSY/FPY/MSY): 26,057	Visual scores (1–6) at weaning: BSW (body structure), FPW (finishing precocity), MSW (muscling); at yearling: BSY, FPY, MSY; body weight at 210 d (W210) and 450 d (W450)	Model selection by AI-REML (WOMBAT); final single- & two-trait Bayesian analyses (THRGIBBS1F90); VS analyzed with linear models; maternal genetic & permanent env. modeled for weaning traits	Two-trait average h²: BSW 0.28, FPW 0.30, MSW 0.27, W210 0.28, BSY 0.40, FPY 0.44, MSY 0.39, W450 0.50	High positive r^g across scores/ages (≈0.40–0.96). Examples (r^g): BSW–W210 0.86, BSW–BSY 0.96, FPW–FPY 0.96, MSW–MSY 0.94, W210–W450 0.96; VS with W450 typically 0.73–0.91	—
**Viana et al. (2020)**	W550 44,372; D400 43,779; BS550 1,745; FP550 1,745; MS550 1,745; SC550 5,524; AFC 13,725	Post-yearling: W550 (weight @ 550 d), D400 (days to 400 kg), visual scores BS550/FP550/MS550 (1–6), SC550; female AFC	Single-trait AIREML (WOMBAT) for linear traits; threshold (Bayesian) animal model for visual scores; PCA on EBVs to build state/sex selection indices	W550 0.32 ± 0.01; D400 0.39 ± 0.01; BS550 0.20 ± 0.06; FP550 0.37 ± 0.08; MS550 0.44 ± 0.08; SC550 0.51 ± 0.05; AFC 0.05 ± 0.06	Reported EBV correlations include W550–D400 = −0.85 (opposite quadrants in PCA); SC550–AFC = −0.89 (males vs. females); visual scores mutually high (≈0.91–0.94).	—

**Regatieri, Boligon & Albuquerque (2011)**	Weaning VS: 79,884; Yearling VS: 53,866; Mature cow weight: 3,260	Visual scores at weaning (205 d) and yearling (550 d): Conformation (C), Finishing precocity (P), Muscling (M); Mature female weight (MW)	Multi-trait REML (WOMBAT) animal model; fixed CG + age covariates (animal & dam; dam age not for MW); random direct additive; maternal additive + maternal permanent env. for weaning scores	Weaning: C 0.18 ± 0.02, P 0.21 ± 0.01, M 0.22 ± 0.01; Yearling: C 0.24 ± 0.01, P 0.27 ± 0.01, M 0.26 ± 0.01; MW: 0.42 ± 0.02	Weaning↔Yearling (same score): 0.56–0.85. VS↔MW: Weaning C/P/M 0.21/0.24/0.27; Yearling C/P/M 0.30/0.33/0.35 (all positive).	—
**Vargas, G. (2018)**	355.524 — 3,519 (HD and imputed; 471,880 SNPs after QC)	Growth, visual scores (conformation, precocity, muscling), and reproductive traits (analyzed via PCs)	PCA on multi-trait additive (co)variance matrix; GWAS on PCs using wssGBLUP and BayesC; haplotype-block windows; genomic prediction with BLUPF90/GS3	Not directly estimated in this thesis (focus is on PCs & genomic prediction)	Not reported (PC-based analysis)	Multiple autosomes; top windows per PC (growth/biotype/weaning–yearling axes) — Not itemized in text summary; genes from top-10 windows were taken to functional analysis
**Santana Jr., Eler, Cucco, Bignardi & Ferraz (2013)**	Pedigree: 167,854; Records: HH 96,876, PRE 114,013, MUS 117,846, BONE 40,844, HP 28,887 (yearlings; HP at ~14 mo)	Hip height (HH, cm); visual scores at ~18 mo: finishing precocity (PRE, 1–6), muscling (MUS, 1–6), bone (BONE, 1–3); heifer pregnancy at 14 mo (HP, 0/1)	Bayesian multi-trait linear–threshold animal model (THRGIBBS1F90); CG, age at measure, dam age effects; Gibbs sampling, HP & BONE on threshold scale	HH 0.36 (0.35–0.38); PRE 0.26 (0.25–0.28); MUS 0.26 (0.25–0.28); BONE 0.26 (0.23–0.29); HP 0.50 (0.45–0.56)	Highlights (posterior means): PRE–MUS 0.99; PRE–BONE 0.85, MUS–BONE 0.85; HH–PRE 0.25, HH–MUS 0.21, HH–BONE 0.38; HH–HP −0.06; PRE–HP 0.25, MUS–HP 0.24; BONE–HP 0.03. Residual r generally low; PRE–MUS residual 0.76.	—
**Raidan et al. (2017)**	Total bulls: 33,013 (538 pasture tests; 213 feedlot tests). Per trait/environment: FW 24,910 (pasture), 8,103 (feedlot); ADG 24,910 (pasture), 8,103 (feedlot); SC 14,888 (pasture), 4,676 (feedlot); VS 23,509 (pasture), 8,046 (feedlot).	Final weight (FW), average daily gain (ADG), scrotal circumference (SC), composite visual score (VS = sum of E, P, M, U, R, A, S; with navel (U) recoded so higher = better).	Bayesian multi-trait animal model (8 traits), Gibbs sampling (GIBBS1F90). Fixed: test effect + linear regression on final age (nested in test). Random: direct additive and residual. Separate (co)variance structures by environment.	Pasture: FW 0.46, ADG 0.25, SC 0.56, VS 0.31. Feedlot: FW 0.49, ADG 0.25, SC 0.60, VS 0.42 (posterior means; HPD90 in paper).	Within environment (pasture / feedlot): FW–ADG 0.74 / 0.67; FW–SC 0.49 / 0.60; FW–VS 0.79 / 0.85; ADG–SC 0.37 / 0.34; ADG–VS 0.65 / 0.74; SC–VS 0.46 / 0.53. Across env. (pasture ↔ feedlot same trait): FW 0.74, ADG 0.44, SC 0.84, VS 0.71.	—
**Soares, Brunes, Baldi, Carmo, Narciso, V.S. Magnabosco, Sainz & C.U. Magnabosco (2023)**	Visual scores: 4,175; Repro: SC365 1,065; SC450 1,053; STAY 932; PPC30 2,102; AFC 3,075	Visual scores (MERCOS): Muscularity (M), Physical structure (PS), Racial aspects (R), Conformation (C), Navel/Omphalos (N), Sacrum (SAC); Reproductive: SC365, SC450, STAY, PPC30, AFC	Bayesian animal models (THRGIBBS1F90): threshold for VS, PPC30, STAY; linear for SC365, SC450, AFC; two-trait analyses; CG fixed effects; age covariates; no maternal/PE (≤3%)	VS h^2 (mean±SD): M 0.21±0.01, PS 0.28±0.01, R 0.25±0.01, C 0.25±0.01, N 0.15±0.01, SAC 0.18±0.01. Repro h^2: SC365 0.50±0.01, SC450 0.53±0.01, STAY 0.38±0.02, PPC30 0.54±0.02, AFC 0.10±0.01.	Selected r^g (VS↔repro): N–STAY −0.25±0.29; N–AFC −0.24±0.27; N–SC365 0.08±0.21; N–SC450 −0.06±0.18. Also: C–SC365 0.25±0.22; C–STAY 0.36±0.25; SAC–STAY 0.66±0.31; SAC–PPC30 −0.55±0.23.	—
**Vargas et al. (2018)**	Animals: 355,524. Per trait (N): BWG 334,123; WC/WP/WM 334,123; WYG 141,131; YC/YP/YM 165,287; SC 58,912	Growth: BWG, WYG; Visual scores (1–5 within CG) at weaning WC, WP, WM and yearling YC, YP, YM; Repro: SC	Multi-trait animal model (WOMBAT AIREML); weaning traits included direct, maternal genetic, and maternal permanent env. effects; yearling traits direct only. PCA on: (i) eigen-decomposition of additive	BWG 0.17±0.01; WC 0.17±0.01; WP 0.21±0.01; WM 0.20±0.01; WYG 0.18±0.01; YC 0.34±0.01; YP 0.34±0.01; YM 0.34±0.01; SC 0.46±0.02	All positive; within-age: weaning 0.39–0.96, yearling 0.25–0.94; across ages: 0.15–0.86 (e.g., BWG with WC/WP/WM 0.52–0.88; WYG with YC/YP/YM 0.49–0.57).	—

	genetic (co)variance (AT); (ii) (co)variance of EBVs (single- and multi-trait).	
